# Exposure to the BPA-Substitute Bisphenol S Causes Unique Alterations of Germline Function

**DOI:** 10.1371/journal.pgen.1006223

**Published:** 2016-07-29

**Authors:** Yichang Chen, Le Shu, Zhiqun Qiu, Dong Yeon Lee, Sara J. Settle, Shane Que Hee, Donatello Telesca, Xia Yang, Patrick Allard

**Affiliations:** 1 Molecular Toxicology Inter-departmental Program, University of California, Los Angeles, Los Angeles, California, United States of America; 2 Department of Integrative Biology and Physiology, University of California, Los Angeles, Los Angeles, California, United States of America; 3 Department of Environmental Health Sciences, University of California, Los Angeles, Los Angeles, California, United States of America; 4 Institute for Society and Genetics, University of California, Los Angeles, Los Angeles, California, United States of America; 5 Department of Biostatistics, University of California, Los Angeles, Los Angeles, California, United States of America; Cornell University, UNITED STATES

## Abstract

Concerns about the safety of Bisphenol A, a chemical found in plastics, receipts, food packaging and more, have led to its replacement with substitutes now found in a multitude of consumer products. However, several popular BPA-free alternatives, such as Bisphenol S, share a high degree of structural similarity with BPA, suggesting that these substitutes may disrupt similar developmental and reproductive pathways. We compared the effects of BPA and BPS on germline and reproductive functions using the genetic model system *Caenorhabditis elegans*. We found that, similarly to BPA, BPS caused severe reproductive defects including germline apoptosis and embryonic lethality. However, meiotic recombination, targeted gene expression, whole transcriptome and ontology analyses as well as ToxCast data mining all indicate that these effects are partly achieved via mechanisms distinct from BPAs. These findings therefore raise new concerns about the safety of BPA alternatives and the risk associated with human exposure to mixtures.

## Author Summary

The intricacy of dealing with chemical alternatives is particularly salient in the case of Bisphenol A which was largely driven out of commercial products in North America by consumer and NGO pressure. However, when choosing chemicals with similar physical attributes, substitutes are most often chosen among chemicals with related structural properties. Therefore, it is essential to carefully assess in which ways the substitute differ enough from the original chemical so as not to elicit the same biological responses while conserving the desirable physical characteristics. Here, we show that both BPA and its common substitute BPS elicit a strong negative effect on germline function in a canonical model for the study of germ cell differentiation: the nematode *C*. *elegans*. However, by performing and analyzing multiple targeted as well as unbiased gene expression studies, we show that, unexpectedly, these reproductive defects are mainly achieved through independent genetic responses. We further validated these results in mammals by performing an in-depth analysis of curated mammalian toxicity databases (ToxCast, Tox21) containing over 125 *in vitro* assays for 4 BPA substitutes. Our results highlight the need for coordinated safety assessment of multiple substitutes and mixtures of these chemicals before their use in product replacement.

## Introduction

Clearly delineating the effects of environmental chemicals on biological processes is a fundamental challenge with direct relevance to public health. The toxicity testing of Bisphenol A (BPA), a common plastic manufacturing chemical, has highlighted the difficulty in determining chemical safety across a wide array of exposure levels and biological endpoints [1–7]. BPA possesses a weak estrogenic activity, and its human exposure is often considered too low to cause deleterious effects [2]. However, the description of low and ultra-low dose responses, the identification of new exposure routes, such as transdermal exposure, combined with significant amounts of BPA in thermal paper have all renewed the concerns for its safety and the impetus for its substitution with less toxic alternatives [5, 6, 8].

An important concern with BPA is its ability to alter germ cell and reproductive function. In particular, rodent exposure to physiologically relevant doses of BPA (approximately 400 ng/day) has revealed two windows of germ cell sensitivity [9]. Exposed adult female mice display chromosome congression failure at metaphase I and II, leading to chromosome segregation errors at anaphase and the generation of aneuploid oocytes [10]. *In utero* exposure, on the other hand, impacts the early stages of germ cell meiotic differentiation in rodents as well as in rhesus monkeys [9, 11]. Specifically, the process of synapsis, or the physical linkage of homologous chromosomes during pachytene I, is impaired as revealed by the presence of partially synapsed homologues [9]. Furthermore, the associated process of meiotic recombination is also altered as shown by an increase in homologous crossover frequency [9]. The alteration of these two fundamental meiotic processes correlates with an increased incidence of chromosome end-to-end associations as well as oocyte and embryonic aneuploidy [9, 11].

These results were further validated in a third species: the nematode *Caenorhabditis elegans (C*. *elegans)*. The nematode is a model organism commonly used for the genetic analysis of meiotic processes [12, 13]. It not only offers great ease of manipulation but also a vast array of tools for the mechanistic dissection of the highly conserved germline and toxicity pathways [14]. In *C*. *elegans*, BPA exposure impairs the kinetics of double-strand break repair inherent to homologous recombination. This effect is associated with abnormal chromosome morphogenesis at the end of prophase I, the generation of oocytes with frayed chromosomes, chromosome segregation errors and embryonic aneuploidy [15, 16]. These results therefore suggested that the pathways required for normal germline maintenance altered by BPA exposure are evolutionary conserved.

4,4′-Sulfonyldiphenol or Bisphenol S (BPS) is a structurally-related and increasingly prevalent substitute for BPA in “BPA-free” products, found at a level higher than that of BPA in thermal paper [17]. In countries where BPS has been used for over a decade as a BPA substitute in aluminum can and paper production, the estimated human exposure level of BPS has already surpassed that of BPA [18]. While our understanding of BPS toxicity is still limited, recent evidence suggest that BPS, akin to BPA, is able to induce alterations in embryonic nervous and endocrine systems [19–21]. Furthermore, several reproductive studies using zebrafish have described a sharp decrease in production of male and female gametes [20, 22] although the mechanism of action of BPS for this endpoint remains to be elucidated.

Here, we present a comparative analysis of BPA and BPS interaction with the meiotic program in *C*. *elegans*. We report that BPS exposure, similarly to BPA, significantly reduces the nematode’s fertility and impairs proper homologous chromosome synapsis during meiotic differentiation, which in turn induces germline checkpoint activation and germline apoptosis. However, while BPA alters the turnover of meiotic recombination factors in late pachytene, BPS does not. Targeted quantitative gene expression studies as well as unbiased transcriptomic analysis and ToxCast data analysis revealed that BPS and BPA trigger primarily distinct transcriptional and biological responses although, in the *C*. *elegans* germline, their respective exposures both converge on effectors of germline checkpoints. These results therefore suggest that BPS may not represent a safe alternative to BPA with regards to reproductive and germline toxicity.

## Results

### *C*. *elegans* exposure conditions to Bisphenol A and Bisphenol S

We characterized and compared the effects of Bisphenol A and Bisphenol S on the nematode’s reproductive processes by following an exposure protocol similar to the one previously described [16]. Briefly, *C*. *elegans* nematodes were exposed for four days, from the first larval stage until the completion of the first day of adulthood (24 hours post-L4 stage) to several concentrations of BPA and/or BPS to establish dose responses: 125 μM, 250 μM and 500 μM. Because of the worm’s cuticle acting as a potent barrier, the lethal dose 50 (LD50) for BPA and BPS in *C*. *elegans* larvae and adults is high, at 1.7mM [23]. No lethality or overt toxicity on morphology or behavior were detected on the worms exposed to the much lower concentrations used in this study. These external concentrations were chosen to generate a range of internal doses in *C*. *elegans* which would approximate organ BPA levels in human reproductive and fetal tissues such as placental (up to 0.105 μg/g) [24] and umbilical cord samples (up to 0.015 μg/g) [25, 26]. To measure the total internal dose corresponding to the aforementioned media concentrations of BPA and BPS, we established a silyl-derivatization coupled with gas chromatography tandem mass spectrometry (GC-MS) method (see methods section). Following exposure, worms were thoroughly washed and lysed by mechanical homogenization. While we could not measure the intra-gonadal levels of the bisphenols, the removal of the worm cuticle compartment by centrifugation after homogenization allowed us to measure the internal concentration of each compound. We measured the mean internal doses to be 0.23 μg/g (+/- 0.02), 0.68 μg/g (+/- 0.17) and 1.89 μg/g (+/- 0.32) for BPA media concentrations of 125, 250 and 500 μM, respectively (S1 Table). For BPS, the media concentrations of 250 and 500 μM translated into the respective internal concentrations of 0.21 μg/g (+/- 0.01) and 0.39 μg/g (+/- 0.01). The media concentration of 125 μM BPS yielded an internal dose that fell below the sensitivity threshold of our approach of 0.1 μg/g. The imbalance in the internal *C*. *elegans* concentrations of BPA and BPS exposure mirrors the levels and ratios in human serum in the United States, where BPA levels are 3 to 5 times higher than those of BPS: 1.36 μg/g creatinine-adjusted for BPA and 0.304 μg/g creatinine-adjusted for BPS [18, 27, 28]. Taken together, we have identified exposure and dose conditions in *C*. *elegans* that approximate human internal physiological concentrations of BPA and BPS.

### Fertility assessment following exposure to BPA and BPS

**Fig 1:**
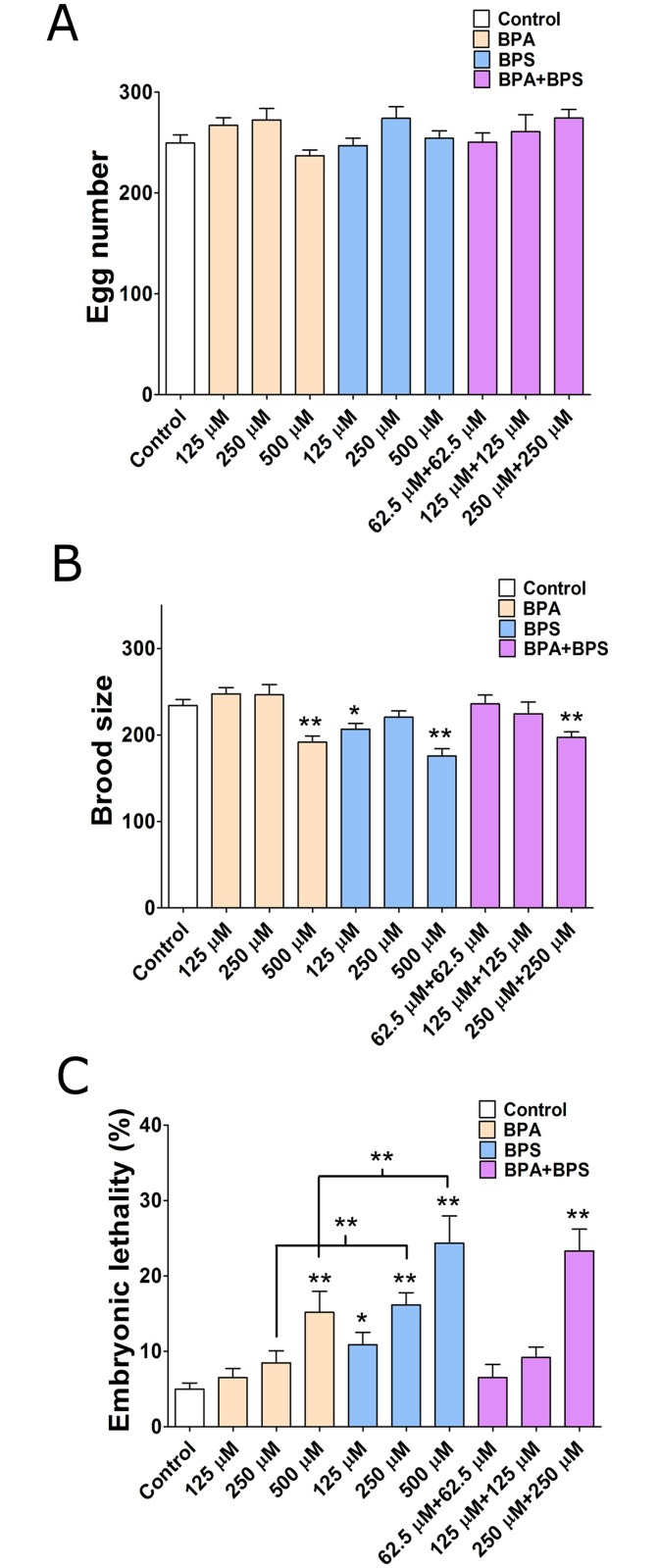
Bisphenols exposure results in sterility, reduced brood size and increased embryonic lethality in *C*. *elegans*. Reproductive response to treatment with either vehicle (0.1% ethanol) or indicated doses of BPA and/or BPS. (A) Average number of eggs laid by singled hermaphrodites in each treatment group. (B) Average number of adult progeny (brood size) from singled hermaphrodites in each treatment group. (C) Embryonic lethality observed among the progeny of hermaphrodites in each treatment group. N = 3–4 worms per trial, three repeats per treatment group. All tests are based on t statistics, calculated at each exposure concentration level. *P<0.05, **P≤0.01.

To assess the impact of BPA and BPS exposures on fertility, we followed nematodes exposed to BPA, BPS or a combination of BPA and/or BPS for the duration of their reproductive period. Three concentrations of the chemicals were used either alone or in combination and each was compared to vehicle control ethanol (0.1% final concentration). The total number of embryos, larvae and adults were tallied over the length of the reproductive period and the brood size (total number of adults), viable egg number and rate of embryonic lethality were calculated. As shown in Fig 1A, the number of fertilized eggs produced by worms exposed to all concentrations of either BPA, BPS or a combination of BPA and BPS did not significantly differ from control but showed a trend towards elevated embryo number at intermediate concentrations as previously shown for BPA, estradiol and several xenoestrogens [29]. However, compared to control, the brood size, was moderately decreased at 500 μM of either BPA (-18%, P*<*0.01) or BPS (-25%, P<0.01) and similarly decreased by exposure to the BPA and BPS mixture (250+250 μM) (-17%, P<0.01) (Fig 1B). The investigation of the cause of the decrease in brood size revealed a corresponding dose-response elevation in the rate of embryonic lethality. At the highest concentration of 500 μM, there was a 3-fold increase in BPA-induced lethality compared to the control, a further 5-fold increase for BPS and near 5-fold for the mixture (Fig 1C). Interestingly, the increase in embryonic lethality was significant for all BPS doses tested. Together, these experiments demonstrate that, as with BPA, BPS causes a sharp decrease in brood size originating from a decrease in embryonic viability at the highest BPS concentration or when combined with BPA.

### BPS elicits germline structural aberrations and chromosome morphogenesis defects

**Fig 2:**
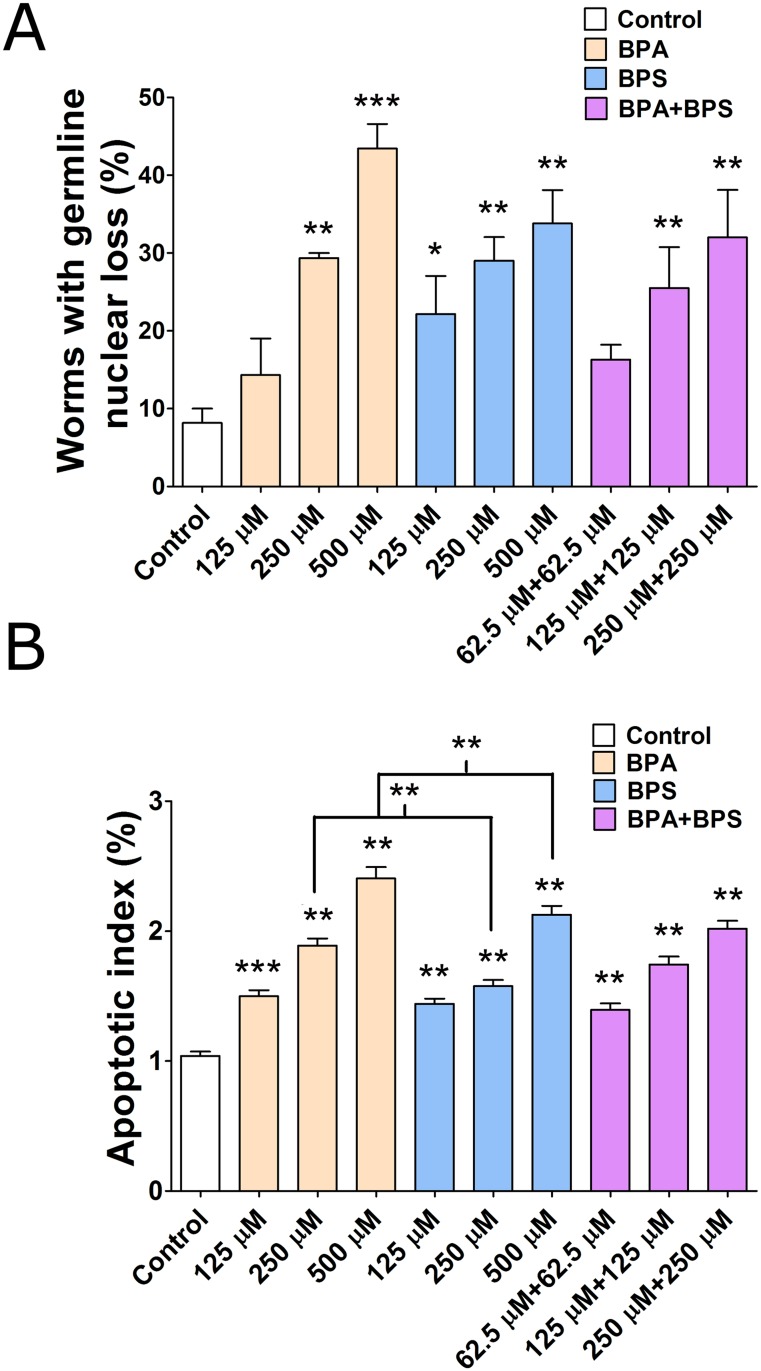
Bisphenols exposure induces germline nuclear loss and apoptosis. (A) Quantification of germline nuclear loss frequency in the gonad of worms exposed to vehicle or to the indicated doses of BPA and/or BPS. N = 20 worms per trial, three repeats per treatment group. (B) Apoptotic index, i.e. the number of apoptotic nuclei number per 100 pachytene nuclei in the gonad of worms exposed to vehicle control (0.1% ethanol) or to the indicated doses of BPA and/or BPS. N = 20 worms per trial, four repeats per treatment group. Error bars represent SEM. All tests are based on t statistics, calculated at each exposure concentration level *P<0.05, **P<0.01 and ***P≤0.001.

The reduction in embryonic viability may stem from the dysregulation of germline function, which is associated with elevated rates of aneuploidy and embryonic lethality [16, 30, 31]. To assess whether the reduction in fertility originates from germline errors, we first examined the overall morphology of the germline and of its nuclei by DNA staining of worms exposed to BPA, BPS or a mixture of both. Consistent with previous observations [16], at the highest concentration, BPA caused a significant 18% reduction in overall germline size as estimated by nuclear count when compared to the ethanol control (P<0.001, N = 15–20) while BPS treated worms displayed a non-significant trend towards a larger germline (S1 Fig). In contrast to the distinct effect of the Bisphenols on the size of the germline, both BPA and BPS exposure caused an irregular distribution of germline nuclei along the distal to proximal axis. Specifically, gaps were apparent in the mid- to late-pachytene stages, suggesting a localized nuclear loss. Quantification of the nuclear gaps frequency also showed a dose-response with a stronger effect for BPA than for BPS (Fig 2A and S2A Fig). The germline nuclear gap phenotype was observed at the highest bisphenols concentrations in 43% of the worms exposed to BPA (P<0.001), 34% of the worms exposed to BPS (P<0.01) and 32% of the worms exposed to the mixture (P<0.05), in contrast to only 10% of control worms. Furthermore, the size of the pachytene gaps in BPA exposed animals was consistently larger than in the BPS group (S2A Fig).

We previously correlated the observed germline size defects following BPA exposure with aberrant kinetics of chromosome morphogenesis at the stage of diakinesis (late prophase I). Specifically, we observed that chromosomes failed to condense properly prior to fertilization and displayed a fragile appearance ([16] and S3 Fig). We interpreted these defects as resulting from the smaller germline and shorter diakinesis stage. However, examination of BPS exposed diakinetic chromosomes revealed that chromosome morphogenesis is also abnormal despite normal germline size (S3A–S3C Fig). The failure of diakinetic chromosomes to properly condense was apparent in the -1 oocyte (the oocyte most proximal to the spermatheca) but was particularly pronounced and significant in the -2 and -3 oocytes. Furthermore, the diakinetic phenotype was concentration dependent and tracked with the observed embryonic lethality (Fig 1C). The impact on the kinetics of chromosome morphogenesis was confirmed by immunostaining diakinetic nuclei with the Synaptonemal Complex (SC) component SYP-1, which under normal conditions, shows a gradual disassembly as the oocytes progress through diakinesis and distinct chromosomal domains are established [32]. We observed a significant alteration of SYP-1 disassembly following BPA, BPS and BPA+BPS exposure. These results showed that both bisphenols induce germline structural defects and that their chromosome morphogenesis phenotype is independent of their effect on germline size (S3D and S3E Fig).

### BPS causes a dose-dependent increase in germline apoptosis and checkpoint activation

The location of the observed germline gaps coincides with the stage at which germline apoptosis occurs in response to a variety of signals, such as the presence of unrepaired or aberrant homologous recombination intermediates and synapsis defects [33–35]. In order to determine whether BPA and BPS cause germline apoptosis, we stained engulfed germline nuclei in exposed living worms with acridine orange [36]. We observed a concentration-dependent increase in germline apoptosis for BPA, BPS and their mixture (Fig 2B and S2B Fig). The nuclear loss and increased apoptosis may stem from an earlier impact on the mitotic region of the germline that then impedes meiotic differentiation. To address the possibility of DNA damage induction and mitotic errors following exposure, we measured the diameter of mitotic nuclei as checkpoint-dependent cell cycle arrest and mitotic nuclear enlargement is a commonly observed feature in repair defective mutants or following exposure to DNA damaging agents [37–40]. The mean nuclear diameter following exposure to BPA or BPS is indistinguishable from control suggesting that these chemicals do not cause observable DNA damage (S4 Fig).

**Fig 3:**
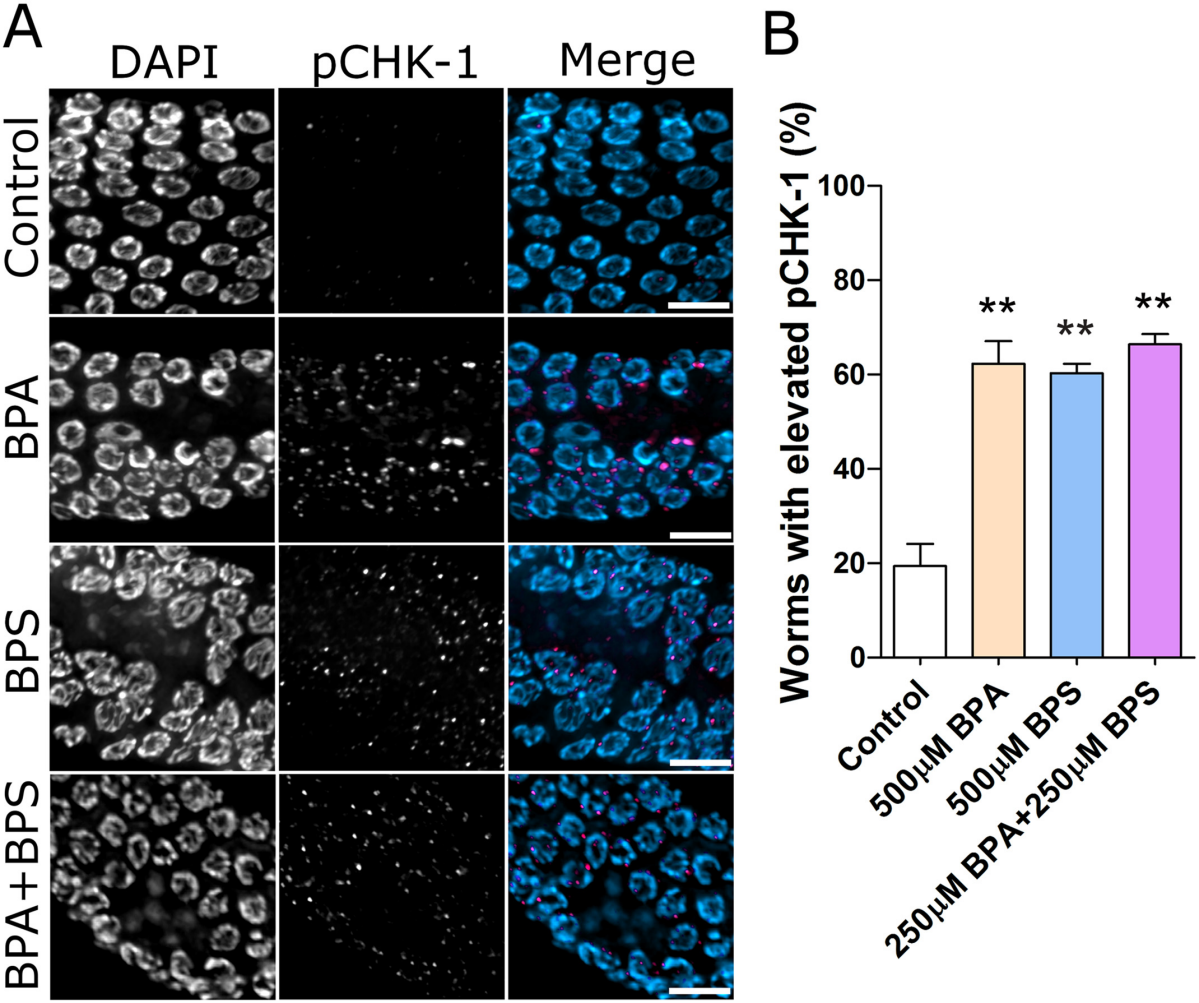
Bisphenols exposure induces DNA damage checkpoint kinase CHK-1 activation. (A) Immunostaining of phosphorylated CHK-1 on mid- to late-pachytene nuclei from dissected gonads of worms exposed to vehicle control (0.1% ethanol), 500 μM BPA,500 μM BPS or to their mixture (Scale bar, 10 μm). (B) Percentage of examined worms with elevated pCHK-1 in each group. Error bars represent SEM. N = 10 worms per trial, three repeats per treatment group. All tests are based on t statistics. **P<0.01.

BPA-induced germline apoptosis is in part mediated by the activation of the pCHK-1/CEP-1(p53) axis in response to aberrant meiotic recombination [16]. CHK-1, a checkpoint protein activated in response to single or double-strand breaks [41], is dramatically activated (i.e. phosphorylated) following BPA exposure [16]. We therefore compared the incidence of elevated pCHK-1 in the germline of worms exposed to BPA and/or BPS. Similar to BPA, we observed a significant up-regulation of pCHK-1 staining following BPS and BPA+BPS exposures (Fig 3A and 3B). Furthermore, we investigated whether CHK-1 activation and the observed regions of nuclear gaps in the germline were correlated events. The vast majority of germlines positive for pCHK-1 in all bisphenol treatment groups also displayed gaps (S5 Fig). Conversely, only a few germline, less than 20%, either exhibited gaps or were positive for pCHK-1 suggesting that pCHK-1 activation and nuclear loss are linked events.

Next, we verified that the observed induction in apoptosis is mechanistically and causatively related to the activation of the DNA damage response (DDR) checkpoint activation. To this aim, we measured the apoptotic index in wildtype (N2) worms as well as in a *spo-11* mutant background where double-strand breaks are not initiated [42], as well as in four additional mutant backgrounds: *cep-1* (worm p53 homologue), *hus-1*, *clk-2* and *chk-1*, genes that cooperate in the establishment of the DNA damage checkpoint response [36]. In all cases, the increased apoptotic levels observed following bisphenol exposure was absent (S6 Fig) indicating that both BPA and BPS exposures elicit a DNA damage checkpoint response.

Together these results indicate that a profound perturbation of the germ cell differentiation processes following exposure to BPA and/or BPS causes the activation of the DNA damage checkpoint.

### BPA, but not BPS, delays the kinetics of meiotic recombination

**Fig 4:**
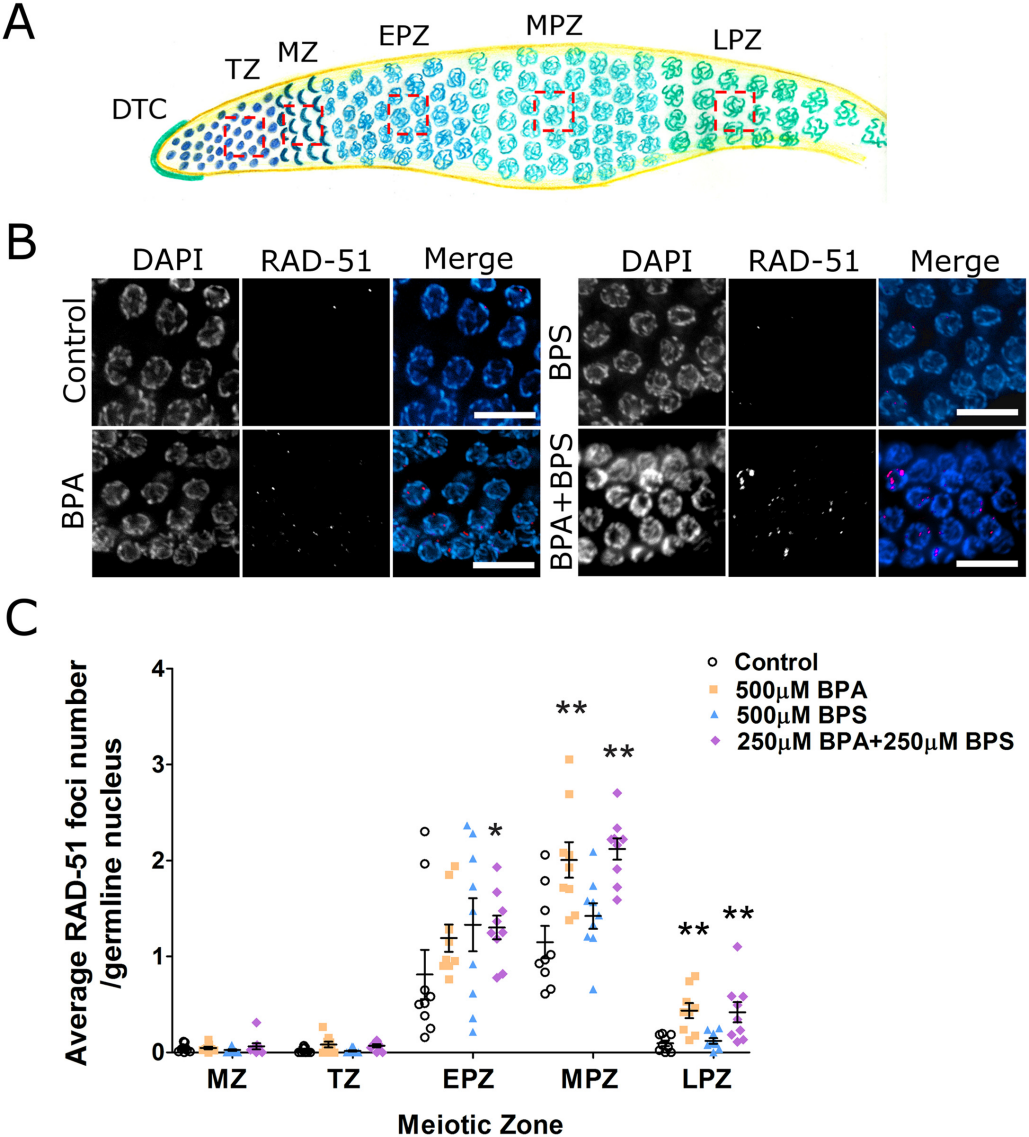
BPS does not alter the turn-over of DSBR protein RAD-51 in late pachytene. (A) Schematic representation of each meiotic stage in *C*. *elegans* germline. Each red square represents the area used for scoring RAD-51 foci. MZ: mitotic zone, TZ: transition zone, EPZ: early pachytene zone, MPZ: middle pachytene zone, LPZ: late pachytene zone. (B) Immunostaining of LPZ RAD-51 foci exposed to vehicle control (0.1% ethanol), 500 μM BPA, 500 μM BPS or to their mixture (250 μM BPA + 250 μM BPS). RAD-51 levels in LPZ are elevated in BPA and mixture but not BPS exposed or control group (scale bar, 10μm). (C) Quantitation of RAD-51 foci per germline nuclei of vehicle (0.1% ethanol) and bisphenols -exposed gonads. The average number of RAD-51 foci per nucleus with SEM (y axis) in each prophase I meiotic stage (x axis). N = 3 worms per group, three repeats per treatment group. All tests are based on t statistics. *P<0.05 and **P<0.01.

We and others have shown that BPA exposure causes a profound alteration of the process of meiotic recombination in a variety of model systems including mice, rhesus monkeys and *C*. *elegans* [9, 11, 16]. In particular, the kinetics of double-strand break repair by homologous recombination, a process inherent to the meiotic program in all sexually reproducing organisms, is severely impacted by BPA as evidenced by defective turn-over of the single-strand invasion factor RAD-51 [16]. We observed a significant elevation of RAD-51 levels following BPA and BPA+BPS exposure in mid-pachytene (Fig 4). As previously observed, a significant proportion of late pachytene nuclei in the BPA group still retained detectable levels of chromatin-associated RAD-51 compared to the ethanol control. Surprisingly, BPS exposure did not significantly delay the kinetics of RAD-51 turnover and its levels in late pachytene were indistinguishable from the control suggesting that BPA and BPS deregulation of double-strand break repair may be mediated, at least in part, by different mechanisms. Finally, BPA and BPS co-exposure induced the same degree of RAD-51 increase as BPA at all meiotic stages, even though the BPA concentration in mixtures is only 250 μM, suggesting a possible synergistic effect with BPS on RAD-51 turnover kinetics.

**Fig 5:**
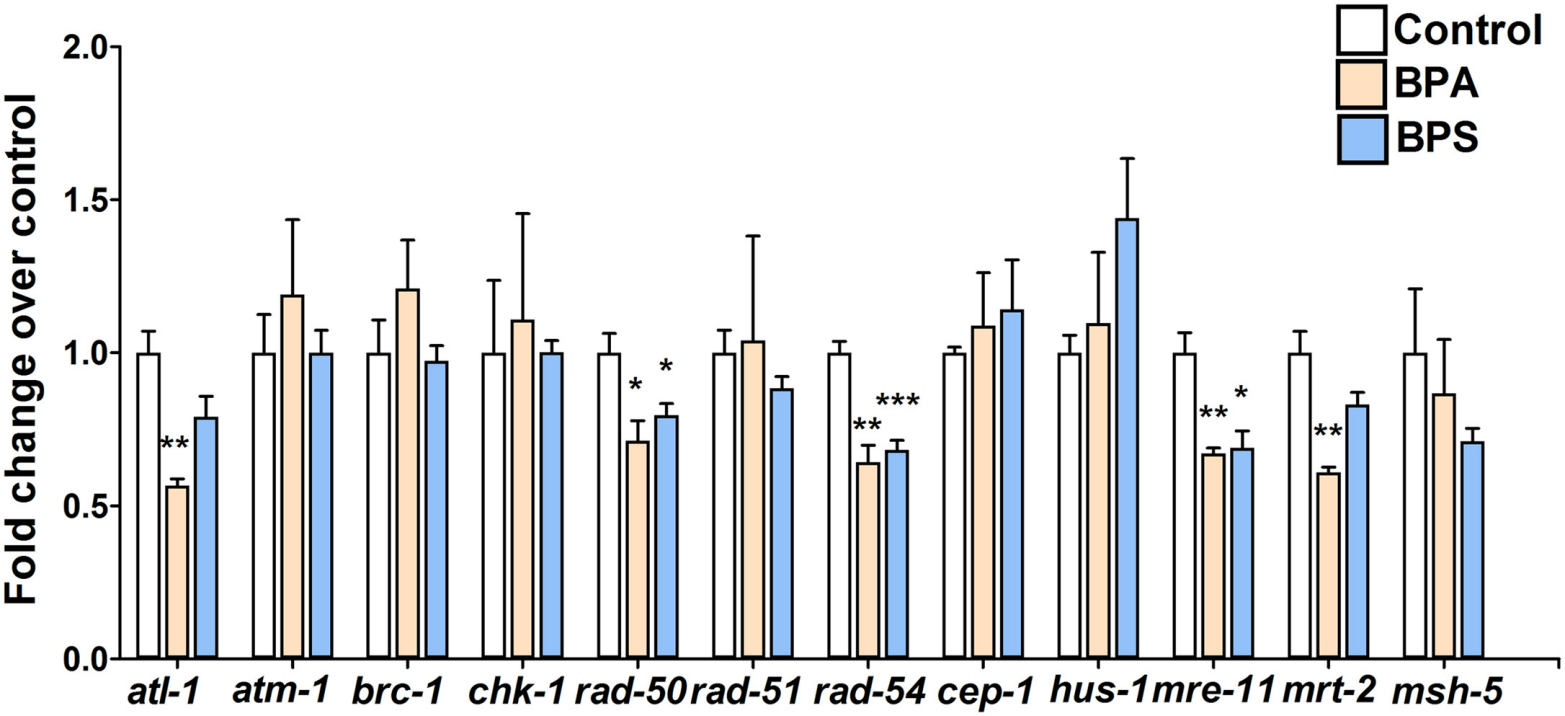
Distinct expression changes of genes implicated in DSBR and DNA damage checkpoints activation pathways. The expression levels of target genes were assayed from isolated germlines by quantitative RT-PCR. Error bars represent SEM for 3–4 biological replicates each tested in duplicate. Two-tailed Student’s *t*-test between vehicle control (0.1% ethanol) and each treatment group (500 μM BPA or BPS). *P<0.05, **P<0.01 and ***P<0.001.

As both BPA and BPS exposures lead to checkpoint activation but only BPA alters the kinetics of RAD-51, we examined other components of the DNA damage repair and checkpoint machinery that may be altered by bisphenols exposure via quantitative reverse-transcription PCR (qRT-PCT) of germline RNA isolated from exposed worms. As previously reported [16], we detected an altered expression of several components of the DNA damage and checkpoint activation pathway following BPA exposure (Fig 5). In particular, we observed a modest but statistically significant decrease in *rad-50*, *rad-54* and *mre-11* expression levels following BPA as well as BPS exposure. In contrast, some genes showed BPA-specific changes including *mrt-2* and *atl-1* which were downregulated following BPA exposure but remained unchanged by BPS. Together with the RAD-51 kinetics results, these findings indicate that while BPA and BPS share some recombination and DDR targets, they also elicit a distinct impact one these pathways.

**Fig 6:**
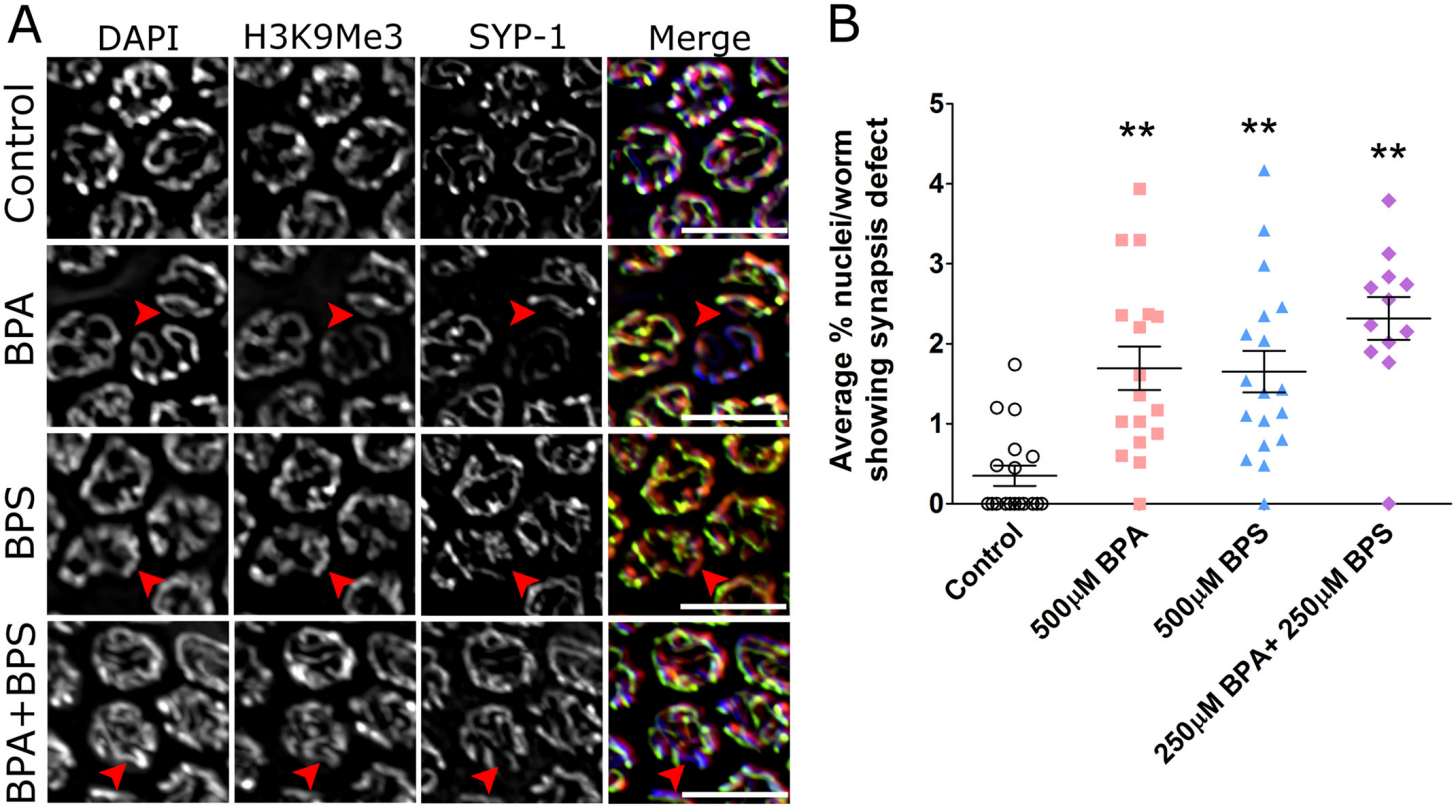
Bisphenol S exposure impairs homologous chromosome synapsis. (A) Partial chromosome synapsis is observed in mid-to-late pachytene nuclei of worms exposed to 500 μM BPA, 500 μM BPS or to their mixture as revealed by SYP-1 (green) and H3K9me3 (red) immunostaining and DAPI (blue). Red arrowheads indicate the chromosomal area lacking SYP-1 expression but displaying H3K9me3 staining. In the BPA exposed germline, one nucleus shows very reduced SYP-1 levels and is likely apoptotic (Scale bar, 10 μm). (B) Average percentage of pachytene nuclei with partial SYP-1 staining for each treatment group. Error bars represent SEM. N = 4–6 worms per trial, three repeats per treatment group. T-test based analysis. **P<0.01.

As checkpoint activation and germline apoptosis are caused either by the presence of aberrant recombination intermediates [36, 40, 43] or of synapsis defects [35], we also examined whether the integrity of the SC was compromised in BPS-exposed nematodes, which could explain the modest but significant elevation of apoptotic nuclei following BPS exposure. To this end, we monitored the assembly and maintenance of the SC by immunofluorescence detection of the central element component SYP-1 [44, 45]. As shown in Fig 6A and 6B, evidence of incomplete synapsis at the stage of pachytene (mid-to-late) was detected following BPA, BPS and BPA+BPS exposures. Quantification of synapsis errors revealed that while a low but significant number of pachytene nuclei in each germline displayed the hallmarks of defective synapsis, these defects were found in almost all (over 90%) of the worms examined. However, as BPA and BPS-induced apoptosis is alleviated in recombination-deficient *spo-11* mutants (S6 Fig), the observed synaptic defects likely stem from the alteration of meiotic DNA repair following exposure.

### BPA and BPS elicit predominantly distinct gene expression signatures

**Fig 7:**
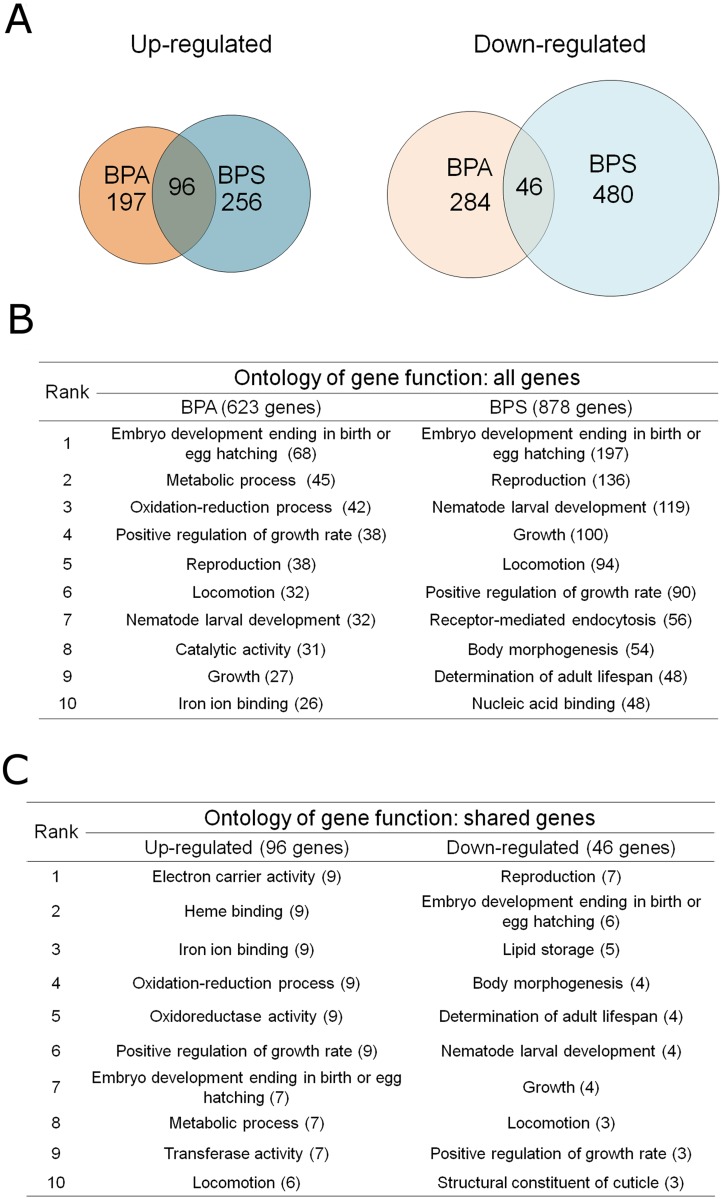
BPA and BPS show distinct alterations of gene expression at whole transcriptome level. (A) Venn diagrams representing the number of genes with a significant (P≤0.05) up- and down-regulation by more than 1.2-fold following exposure to BPA or BPS exposure at 500 μM as assessed by the RNA sequencing analysis of 5 replicates for each treatment group. (B) Functional ontology analysis of all genes identified through the RNA-seq analysis as altered by either BPA or BPS. (C) Functional ontology analysis of shared genes either up- or down- regulated by both BPA and BPS.

In order to further characterize the similarities and differences between the bisphenols’ biological responses, we performed an unbiased assessment of the nematode transcriptome through RNA sequencing (RNA-seq) following exposure to either BPA or BPS, each at 500 μM, in comparison to 0.1% ethanol (control group). At a false discovery rate (FDR)<0.05 after multiple testing corrections, we identified 623 and 878 genes respectively following BPA and BPS exposure that were significantly up- or down-regulated by more than 1.2-fold (Fig 7A). To gain confidence in the validity of the RNA-seq data, we also confirmed the directionality and magnitude of gene expression changes by qRT-PCR for a subset of 20 gene expression/condition relationships causing low, medium or high fold-change values in either direction and observed an 80% concordance in the directionality of expression changes between the two methods (see methods section and S7 Fig). Overall, our RNA-seq analysis revealed that more genes were up-regulated by BPA and BPS than were down-regulated. Surprisingly, the proportion of shared transcripts between BPA and BPS was very low but higher for up-regulated genes (32.7% and 27.2% of the total number of genes up-regulated by BPA or BPS respectively) than for down-regulated genes (13.4% and 8.7% respectively).

To confirm that the divergence in transcriptional response was not unique to *C*. *elegans*, we mined the outcome of 126 *in vitro* tests from EPA’s Toxcast data covering a wide array of transcriptional and biological assays (S8 Fig) [46]. For each assay, we compared the established AC50 for BPS, as well as three other BPA analogues: TGSA, BPF and BPAF, to that of BPA. Our results revealed a low level of biological similarity between BPA and BPS as well as between BPA and TGSA or BPF as indicated by their respective Pearson’s correlation coefficients of 0.68, 0.40 and 0.49. This result contrasted sharply with BPAF which showed a high degree of response similarity to BPA with an r value of 0.85. The Toxcast data therefore indicates that BPA and BPS show a significant divergence in their elicitation of biological pathways.

In order to dissect the functional relevance of the distinct gene expression signatures stemming from BPA and BPS exposure, we conducted gene ontology (GO) analysis on all genes altered by BPA or BPS (Fig 7B and 7C). The GO analysis revealed a convergence on functional categories targeting embryonic development, the top category for both BPA and BPS, and reproduction, ranked 5th for BPA and 2nd for BPS. It also identified distinct functional categories that are not shared between the two chemicals such as metabolic processes (ranked 2nd for BPA) and adult lifespan (ranked 9th for BPS). Importantly, these identified shared functional categories were consistent with our phenotypic analysis of decreased embryonic viability and reproductive function. These results therefore indicate that while the identities of individual genes altered by BPA and BPS are mainly distinct, these genes can be grouped into similar functional categories focused on reproduction and embryonic development.

Finally, as BPA and BPS were found to affect the process of DNA damage repair and recombination in the germline, we tested curated sets of genes known to be involved in these processes to determine whether they were dysregulated by BPA and BPS. We examined the expression changes of a total of 56 DNA repair genes and found that seven of these genes were differentially expressed at a statistical cut-off of P<0.05 in the BPS group. While none reached this cutoff in the BPA group, DNA repair genes showed a highly statistically significant collective bias toward lower P values (i.e., more significant changes) compared to random sets of genes (P<2e-16 by Kolmogorov-Smirnov test; S9 Fig). Therefore, these results indicate that, at internal doses lower than those of BPA, BPS elicits a potent transcriptional response which includes the alteration of DNA damage repair pathways.

## Discussion

We performed a comparative study of the germline response to Bisphenol S and Bisphenol A and showed that both BPA and BPS exposure cause a sharp increase in embryonic lethality and a corresponding decrease in fertility in the nematode *C*. *elegans*. Within the germline, both bisphenols elicited an apoptotic response resulting in germline nuclear loss in late pachytene. However, the impact of BPA and BPS on the meiotic recombination process was distinct, with only BPA altering the completion of meiotic recombination in late pachytene as observed through the lens of RAD-51 kinetics.

In line with these observations, we used two independent means of gene expression analysis and identified a partially non-overlapping response of DNA damage repair pathways to BPA and BPS exposures. While our qRT-PCR study of germline transcripts showed that most genes were either affected by both BPA and BPS or only affected by BPA, our more comprehensive RNA-seq and GO analyses on whole worms revealed that BPS elicits a stronger reproductive and DNA damage repair gene response than that of BPA, even at a lower internal dose. These results correlated well with the embryonic lethality phenotype where BPS’s effect was significantly stronger than BPA’s. Therefore, these two approaches revealed partially unique signature responses which may explain the potential synergistic effect of BPA and BPS co-exposure on the recombination endpoint.

Interestingly, while BPA and BPS differ in their effects on recombination, both exposures converge on the activation of the CHK-1-dependent checkpoint and a shortened, incomplete diakinesis stage. The diakinetic phenotype is independent of gonadal length and germline nuclei number as it is displayed in both BPA-induced shorter/smaller gonads as well as normal-sized BPS-exposed gonads. It is also distinct from the phenotypes of mutants altering oocyte formation such as those of oocyte maturation defective mutants *cdc-25*.*2* or *wee-1*.*3*, which display endo-mitotic nuclei or early differentiation of the nuclei respectively during diakinesis, something not observed in our context [47, 48]. Instead, gene expression analysis by RNA-seq revealed a strong down-regulation following exposure to BPA and BPS of two genes, *msp-50* and *msp-152*, both implicated in the control of oocyte maturation and embryonic viability [49]. These two genes showed a strong down-regulation of their expression, between 70 to 80%, with a low P value: P<0.02 for BPA and P<0.001 for BPS. These genes function by antagonizing Ephrin signaling thereby promoting MAPK activation and oocyte maturation [50, 51]. The role of these genes in regulating oocyte growth and maturation as well as their described RNAi phenotype of embryonic lethality suggest that the suppression of their expression following BPA and BPS exposure may play a significant role in the observed diakinetic and embryonic effects (S3 Fig). The role of these genes in causing diakinetic defects requires further investigation, however they represent two of only seven genes functionally related to reproduction that are shared between BPA and BPS, a small proportion when compared to the total number of reproductive genes altered by BPA (38 genes) or BPS (136 genes).

Our results therefore reveal drastic differences in gene expression changes elicited by BPA and BPS in *C*. *elegans*. These results are not confined to the nematode as evidenced by the analysis of EPA’s Toxcast data. The non-equivalence of these substitutes in their alteration of the transcriptome is of particular concern as co-exposure may affect multiple pathways that are functionally related leading to a worsening of the phenotype as observed here for the embryonic lethality endpoint.

Taken together, our findings clearly highlight the need for comprehensive testing of Bisphenol A substitutes in functional *in vivo* assays such as those described here in *C*. *elegans* to fully characterize the phenotypic outcomes of exposure for complex biological processes such as reproduction.

## Materials and Methods

### Animals and culture conditions

*C*. *elegans* worms were cultured according to [52] at 20°C on nematode growth medium (NGM) plates without cholesterol. The N2 Bristol strain was used as the wild-type background. The following mutations and chromosome rearrangements were used in this study: LGI, *cep-1(lg12501)*, *hus-1(op241)*; LGIII, *clk-1(qm937)*; LGIV *spo-11(OK79)*, and LGV, *chk-1(tm938)/nT1*. BPA (Sigma Aldrich) and BPS (Sigma Aldrich) were dissolved in 100% ethanol and added to NGM to reach the described final bisphenol concentrations while keeping a 0.1% ethanol final concentration. Equal amounts of BPA and BPS were mixed in the combination group (for example 250 μM mixture exposure = 125 μM BPA + 125 μM BPS). Exposure was carried out from the L1 larval stage to adulthood by placing sodium hypochlorite-treated eggs onto bisphenol and control plates followed by incubation at 20°C for 4 days. For all analyses, worms were collected between 20 and 24 hours post-L4. As there was a slight delay in egg-laying between the treatment groups, we collected the BPS–treated group first (~20 hours post-L4), then ethanol and lastly BPA-treated worms (~24 hours post-L4).

### DAPI analysis and immunostaining

Worms were collected at 20–24 hours post-L4 stage and whole-mount preparation of dissected gonads, DAPI staining, immunostaining, and analysis of stained germline nuclei were performed as described in [53], except for pCHK-1 antibodies, where gonads were fixed with 1% (v/v) formaldehyde. Primary antibodies were used at the following dilutions: goat α-SYP-1, 1:250; mouse α-RAD-51, 1:500; mouse α-H3K9Me3, 1:250 (Active Motif) and goat α-pCHK-1, 1:50 (Santa Cruz). The latter was validated by staining a *chk-1*(tm938) mutant where no staining was observed. Secondary antibodies used were FITC anti-goat and Cy3 anti-mouse, each at 1:500 (Jackson ImmunoResearch).

### Imaging and microscopy

Immunofluorescence images were collected at 0.2 μm z intervals with an Eclipse Ni-E microscope (Nikon) and a cooled CCD camera (model CoolSNAP HQ, Photometrics) controlled by the NIS Elements AR system (Nikon). The images presented are projections approximately halfway through 3D data stacks of the *C*. *elegans* gonads. Images were subjected to 3D landweber deconvolution analysis with the NIS Elements AR analysis program (Nikon).

### Quantitative analysis of germ cell apoptosis

Germ cell corpses were scored in adult hermaphrodites between 20 and 24 hours post-L4 stage, using acridine orange as described in [36]. Briefly, adult worms were incubated in M9 solution with 25 μg/ml acridine orange for two hours at room temperature and then transferred onto NGM plates for 10 minutes recovery. Only worms appearing healthy and moving normally were assayed. The number of apoptotic nuclei in one gonad arm was counted under a fluorescent microscope. Apoptotic index, the number of apoptotic nuclei per one hundred nuclei at the pachytene stage, was used to correct for germline size variation.

### RAD-51 time course analysis

RAD-51 foci in germline nuclei of age-matched (20–24 hours post-L4) hermaphrodites were quantified as described in [16] with the following modification: the germline was divided by stages of meiotic prophase I and only the nuclei in the middle of each stage were scored to account for difference in germline sizes between exposure groups (Fig 4A). The average number of nuclei recorded for each stage per worm were: mitotic zone (69), transition zone (23), early pachytene (59), mid-pachytene (58) and late pachytene (46).

### Gas chromatography-tandem mass spectrometry analysis

Worms exposed to each dose of BPA or BPS from L1 stage to adulthood were collected and washed 10 times for 10 minutes each with M9 buffer. To extract Bisphenols, 100 mg of worms were homogenized in HPLC-grade water and centrifuged at 3,000 g for 5 minutes. The lysate was purified on a methanol-water pre-conditioned Strata-X high performance polymeric reversed phase cartridge (Phenomenex) and dried under a 1 L/min stream of nitrogen at 35°C. The dried residue was redissolved and derivatized at 75°C for 30 minutes with 100 μl N,O-bistrifluoroacetamide (BSTFA) containing 1% trimethylsilyl chloride (Cerilliant) for silylation. The final derivative was dried under a 1 L/min stream of nitrogen and reconstituted with 50 μl acetonitrile. GC-MS analysis was performed on an Agilent 6890N gas chromatograph coupled with an Agilent 5973 network mass selective detector. Silylated 4-cumylphenol was directly derivatized from 4-cumylphenol (Sigma Aldrich) and used as internal standard. Silylated BPA and BPS (Sigma Aldrich) were used as external standard. 3 μl of final derived sample was injected into the GC column in splitless mode at 280°C. The quantifiable limit of detection was 0.001ug/g of worm tissue for silylated-BPA and 0.1ug/g of worm tissue for silylated BPS.

### RNA-seq analysis

For RNA-seq analysis, sodium hypochlorite-synchronized worm populations were exposed to either BPA or BPS (500 μM) from L1 larval stage until adulthood (~24 hours post-L4). Worms were collected, washed 10 times for 10 minutes with M9 buffer and total RNA was extracted with using a RNeasy Micro Kit (Qiagen) according to the manufacturer’s protocol. The purity and concentration of extracted RNA were quantified using UV spectroscopy.

The RNA-Seq analysis was performed at the UCLA Clinical Microarray Core. Briefly, the RNA-seq libraries were prepared following the standard Illumina protocol. Paired-end sequencing at 100 bp length and 30 million coverage was performed on HiSeq 2000 (Illumina Inc, CA, USA). Data quality checks were performed on the Analysis Viewer and demultiplexing was performed with CASAVA 1.8.2 (Illumina Inc, CA, USA).

Paired-end RNA-Seq reads were analyzed using the Tuxedo tool package comprised of Tophat [54], Bowtie [55], Cufflinks, and Cuffdiff [56]. Specifically, we used TopHat2/Bowtie2 which allows alignment across splice junctions to map reads to the *C*. *elegans* genome and to discover transcript splice sites. Cufflinks were then used to assemble the aligned reads onto transcripts, and Cuffdiff was used to compare the aligned reads between the BPA- or BPS-treated group and the control ethanol group to identify genes and gene transcripts that were differentially expressed. Multiple hypothesis testing was corrected using the q value method [57] to estimate false discovery rate (FDR). Genes and transcripts showing differential expression or alternative splicing at P<0.05 were defined as a gene “signature” for further testing of biological pathways. A relatively liberal statistical cutoff was used for signature selection because these downstream analyses did not rely on the accuracy of individual genes but utilized the overall patterns of all signature genes.

### RNA extraction and quantitative PCR

Sodium hypochlorite-treated eggs were placed on cholesterol-free NGM plates with 500 μM BPA or BPS as described above. Total RNA was extracted from adult worms or dissected germlines at 24 hours post-L4 with TRIzol following the manufacturer’s protocol. cDNA was synthesized with qSCript XLT cDNA SuperMix (Quanta Biosciences). qPCR was performed on an Applied Biosystems StepOnePlus realtime-PCR machine with Maxima SYBR Green qPCR Master Mixes (Thermo Scientific). Thermal cycling condition was 1x 95°C, 10 min, followed by 40x 95°C, 15s; 55°C, 30s; 72°C, 30s followed by 72°C, 5min. Melting curve analysis was performed to verify the specificity of the PCR amplicons. Primer sequences are shown in the S2 Table. Genes used for RNA-seq validation were C04G2.9, C40H1.8, *col-133*, *gpx-3*, *ugt-18*, *ugt-36*, Y65B4BR.1, *ttr-44*, Y111B2A.1, *pqn-98*, *dsbn-1*, K08E4.2, *sgca-1*, W08E12.8, C48B4.8, *inx-17*, F58F9.3 and *clec-169*.

### Biological similarity analysis

The biological similarities between BPA and each of its analogues (BPS, BPAF, BPF and TGSA) were determined by the correspondence of their AC50 values in 126 ToxCast assays, covering 77 multiplexed transcription receptor assays (ATG), 4 enzyme cell-free (NVS) assays, 15 HeLa cell based gene expression regulation assays (OT), and 23 Tox21 assays (http://actor.epa.gov/dashboard/). AC50 values, the concentration at which an assay is activated or inhibited by 50% when compared to the control values, were firstly converted to the common logarithm values (Log_10_AC50) and then used to calculate the Pearson correlation coefficient, r value. The biological similarity was judged by the proximity of r value toward 1.

### Statistical analysis of phenotypes

In all assays we considered rigorous detection and elimination of experimental artifacts, including batch and threshold effects. Unless specified otherwise, all *in vivo* outcomes were analyzed using a linear model with fully factorial expansion of group means, making no additivity assumptions across exposure concentration and stressor effects. Formal testing of statistically significant difference between groups exposed to stressors and control groups, as well as comparisons between stressors at the same exposure concentration, are based on t tests for the appropriate contrast in the linear model. Considering our sample sizes, we identified as statistically significant all mean differences with P values below 0.05, controlling the per-hypothesis type I error at 5%.

## Supporting Information

S1 Table Bisphenols internal exposure level as measured by gas chromatography-mass spectrometry. Three biological replicates were subjected to analysis per treatment group.
(PDF)

S2 Table Primer sets used in quantitative RT-PCR experiments. (PDF)

S1 Fig BPA but not BPS exposure results in reduced gonad size. (A) Mean number of germline nuclei in the gonad of worms exposed to vehicle or to the indicated doses of BPA and/or BPS. Error bars represent SEM. More than 5 worms were analyzed per trial and three repeats were performed per treatment group. One-way ANOVA with Tukey’s Post hoc test between control and same treatment with different concentrations. Two-way ANOVA between same concentrion groups of BPA and BPS. ***P<0.001 (B) Low magnification images of DAPI-stained whole-mount gonads from age-matched hermaphrodites exposed to vehicle control (0.1% ethanol), 500 μM BPA, 500 μM BPS or to their mixure (250μM BPA+250μM BPS). (Scale bar, 20 μm).
(TIF)

S2 Fig Bisphenols exposure induces germ cell loss and apoptosis. (A) Low magnification images of DAPI-stained gonads from age-matched hermaphrodites exposed to vehicle control (0.1% ethanol), 500 μM BPA, 500 μM BPS or to their mixture (250μM BPA+250 μM BPS). Red arrows indicate the areas with gaps in gonads which refer to the germ cell loss, N = 20 worms per trial performed in triplicate (Scale bar, 25 μm). (B) Low magnification images of acridine orange-stained apoptotic nuclei in the gonads from age-matched hermaphrodites exposed to vehicle control (0.1% ethanol), 125 μM BPA, 125 μM BPS or to their mixture (62.5 μM BPA+62.5 μM BPS). The highlight spots in each gonad are apoptotic nuclei, N = 20 worms per trial, four repeats per each group (Scale bar, 25 μm).
(TIF)

S3 Fig Bisphenol S exposure impairs chromosome morphogenesis in diakinesis. (A-C) The frequency of -1 to -3 oocytes with chromosomal abnormalities in worms exposed to vehicle control (0.1% ethanol) or to the indicated doses of BPA and/or BPS. The oocyte closest to the spermatheca is referred to as the -1 oocyte. Error bars represent SEM. N = 20 worms per trial performed in triplicate (D) DAPI and SYP-1-stained chromosomes in -1 to -3 oocytes from age-matched hermaphrodites exposed to vehicle control (0.1% ethanol), 500 μM BPA, 500 μM BPS or to their mixture (250 μM BPA+250 μM BPS). Six intact bivalents are observed in the control (vehicle) group. In contrast, chromosomes in oocytes are less defined and organized in the worms treated with Bisphenols and show detectable SYP-1 (green) staining (Scale bar 3 μm). (E) Histogram depicts the frequency of observing SYP-1 expression in -1 to -5 oocytes of worms exposed to vehicle control (0.1% ethanol), 500 μM BPA, 500 μM BPS or to their mixture (250 μM BPA+250 μM BPS). Error bars represent SEM. N = 10 worms per trial performed in triplicate. One-way ANOVA with Tukey’s Post hoc test between control and same treatment with different concentrions. Two-way ANOVA between same concentrion groups of BPA and BPS. *P<0.05, **P<0.01 and ***<0.001.
(TIF)

S4 Fig Diameter of mitotic zone nuclei following exposure. The average diameter of germline nuclei in the mitotic zone from worms exposed to vehicle control (0.1% ethanol), 500 μM BPA or 500 μM BPS was measured. No statistically significant difference was observed between treatment groups and control. Error bars represent SEM. N = 6 worms per trial performed in triplicate. Two-tailed student’s *t*-test between control and each treatment group.
(TIF)

S5 Fig Concordance between germline nuclear loss and pCHK-1 activation. Two events in the germline, nuclear loss and pCHK-1 activation, from worms exposed to vehicle (0.1% ethanol), 500 μM BPA, 500 μM BPS and their mixture were recorded simultaneously. The concordance of pCHK-1 activation and the presence of gaps in all treatments was analyzed by Spearman’s rank correlation test. The correlation coefficient = 0.800, p<0.001, indicating a high positive correlation between these two events. Error bars represent SEM. N = 10 worms per trial, three repeats per treatment group.
(TIF)

S6 Fig Apoptotic response in *spo-11* and DDR mutants. The normalized apoptotic values per 100 pachytene nuclei relative to ethanol control were measured in 6 genetic backgrounds: N2 (wild type), *cep-1*, *hus-1*, *clk-2*, *spo-11* and *chk-1*. In all mutant backgrounds, no statistical difference was observed between BPA or BPS and control except for cep-1 where a small reduction of apoptotic numbers was observed following BPS exposure. N = 13–24 worms per trial performed in triplicate or quintuplate (for N2 and *spo-11* backgrounds). Two-tailed student t-test based analysis, *P<0.05, ***P<0.001.
(TIF)

S7 Fig Concordance of RNA-seq data with qPCR data. Correlation of RNA-seq (y axis) with qPCR data (x axis) using the log2 fold change measure of a total to 20 genes/BPA or BPA exposure tests were analyzed. 16 out of 20 genes showed directional consistency. The Spearman’s rank correlation coefficient = 0.645, p = 0.002, shows a positive correlation between the two data sets.
(TIF)

S8 Fig Biological similarities of BPA analogues to BPA. Biological similarities were determined by the Pearson correlation coefficient, r value, between BPA and each of its analogues using the log_10_AC50 across 126 Toxcast assays. Based on the proximity of r value toward 1, the biological similarity to BPA among four tested analogues is BPAF>>BPS>BPF>TGSA.
(TIF)

S9 Fig Assessment of differential expression of genes involved in DNA repair. We evaluated the overall pattern of differential expression among these DNA repair genes using one-sided Kolmogorov-Smirnov (KS) test and Fisher’s exact test summarized into an enrichment score, defined as log10((PKS+PFisher)/2) to allow comparison of the degrees of enrichment between BPA and BPS. The P value distributions of the DNA repair genes in the BPA and BPS experiments are visualized using q-q plots, where the quantiles of the log-transformed p-values of DNA repair genes (observed) are plotted against the quantiles of the log-transformed P-values of all genes (expected). A shift of the P value distribution to the upper left direction in the q-q plots indicates an overall enrichment of small P values in the DNA repair genes compared to random genes. Dashed red line indicates cut-off P value of P = 0.05.
(TIF)

S1 Data The data used to generate all main and supplementary figures. (ZIP)
