# Supplementary material for: Exposure to the BPA-Substitute Bisphenol S Causes Unique Alterations of Germline Function
Source: PLoS Genet. 2016 Jul 29;12(7):e1006223. doi: 10.1371/journal.pgen.1006223 (PMC4966967; doi:10.1371/journal.pgen.1006223)
Supplement: S1 Table — Three biological replicates were subjected to analysis per treatment group. (PDF) [file pgen.1006223.s001.pdf]

Table S1:

Bisphenols internal exposure level detected by gas chromatography-mass spectrometry

Bisphenol media concentration (μM)	Endogenous Bisphenol concentration (Mean±SD, μg/g worm tissue)	
	BPA	BPS
500	1.89+0.32	0.39+0.01
250	0.68+0.17	0.21+0.01
125	0.23+0.02	<0.100[a]

[a]Under the limit of detection of BPS. N=3 for each group
