# Supplementary material for: Exposure to the BPA-Substitute Bisphenol S Causes Unique Alterations of Germline Function
Source: PLoS Genet. 2016 Jul 29;12(7):e1006223. doi: 10.1371/journal.pgen.1006223 (PMC4966967; doi:10.1371/journal.pgen.1006223)
Supplement: S2 Table — (PDF) [file pgen.1006223.s002.pdf]

Table S2: Primer pairs used for quantitative RT-PCR analysis

	Primer pair	Left Primer sequence	Right primer sequence
1	*atl-1*	gcattctcctgcgttttctc	cgtcgaaccttcgtcttctc
2	*atm-1*	cccgattctgattgaaggaa	ggcttctcggaaatttgtc
3	*brc-1*	caacaatgcggaggagaaat	tacccattccattgtcacca
4	*chk-1*	gtctggtcgtctgggattgt	ttgctgatccatcccatgta
5	*rad-50*	gaaatgcaaacccacgagat	tccattggcttttcgttctt
6	*rad-51*	ccaggctgatgctaaaaagc	ttcggcttctggtaaattgg
7	*rad-54*	cgtcttcgaatgtggatcg	gtcgttttcttcggcttcag
8	*cep-1*	ttccgacgcaagtagtctcc	ccgtttgcattgaacaacac
9	*hus-1*	aagatactgcggcaatcgac	tgaaccaacttccaccatca
10	*mre-11*	ctgtttggaaagcacagcaa	ttgaatgctcgaacaagacg
11	*mrt-2*	tagaaacgggtcaatgcaca	gtgccacgttcctgtatcct
12	*msh-5*	ccccaaaacagctttccata	ggcgtcttgaatggatcact
13	*gpd-1*	actcgtccattttcgatgct	tcgacaacacggttcgagta
14	C04G2.9	cgccaccgaaagaagatact	ctgcttggtggtcagttcag
15	C40H1.8	tcaactgccaacaatgaagc	cctgtccatgagctccctta
16	*col-133*	atgctgtcttccaggagctg	catctccttgtggtcctggt
17	*gpx-3*	ctgcaacggttgatgaaaca	ttgcaacattgacgagaagaa
18	*ugt-18*	aagaaagattttgttggctcaaa	catttcaggatccattctcca
19	*ugt-36*	gtttgttcttcgccctgtgt	cacattatgccctgcgtatg
20	Y65B4BR.1	gagcaaaaataccccgacttc	atgctcgtcgagagagtggt
21	*ttr-44*	tcagtcgattgctgtcaagg	ctccggacaattggaactct
22	Y111B2A.1	ttccacgtgtaccttcacca	tttcaaatcctcctggttgc
23	*pqn-98*	gaagtgcagttggcatcaga	gctggagctgttgatccttc
24	*dsbn-1*	cgtttctgcaaccaaactga	agctgctgatttggcttgtt
25	K08E4.2	atcgacaatcgctcgttctt	cgtccgtctcgattgaaaat
26	*sgca-1*	gaaacattgggcgaattcat	cgcagtggagttcgtgtct
27	W08E12.8	cgctttccatcaaccaactt	gggaaatgcgagagtgttgt
28	C48B4.8	cgtctttgcaatgggataca	tcgtagtagaaagtcggaatgaa
29	*inx-17*	ctgggctccgaaacaattta	tttgttttcccggatagctg
30	F58F9.3	cccatcacaatctgtcagtcc	tgctctgccaatgaaactgt
31	*clec-169*	ttcggaagattttggattgg	ccgcataactgcacacaaac
