# Supplementary figures and images for: Exposure to the BPA-Substitute Bisphenol S Causes Unique Alterations of Germline Function
Source: PLoS Genet. 2016 Jul 29;12(7):e1006223. doi: 10.1371/journal.pgen.1006223 (PMC4966967; doi:10.1371/journal.pgen.1006223)

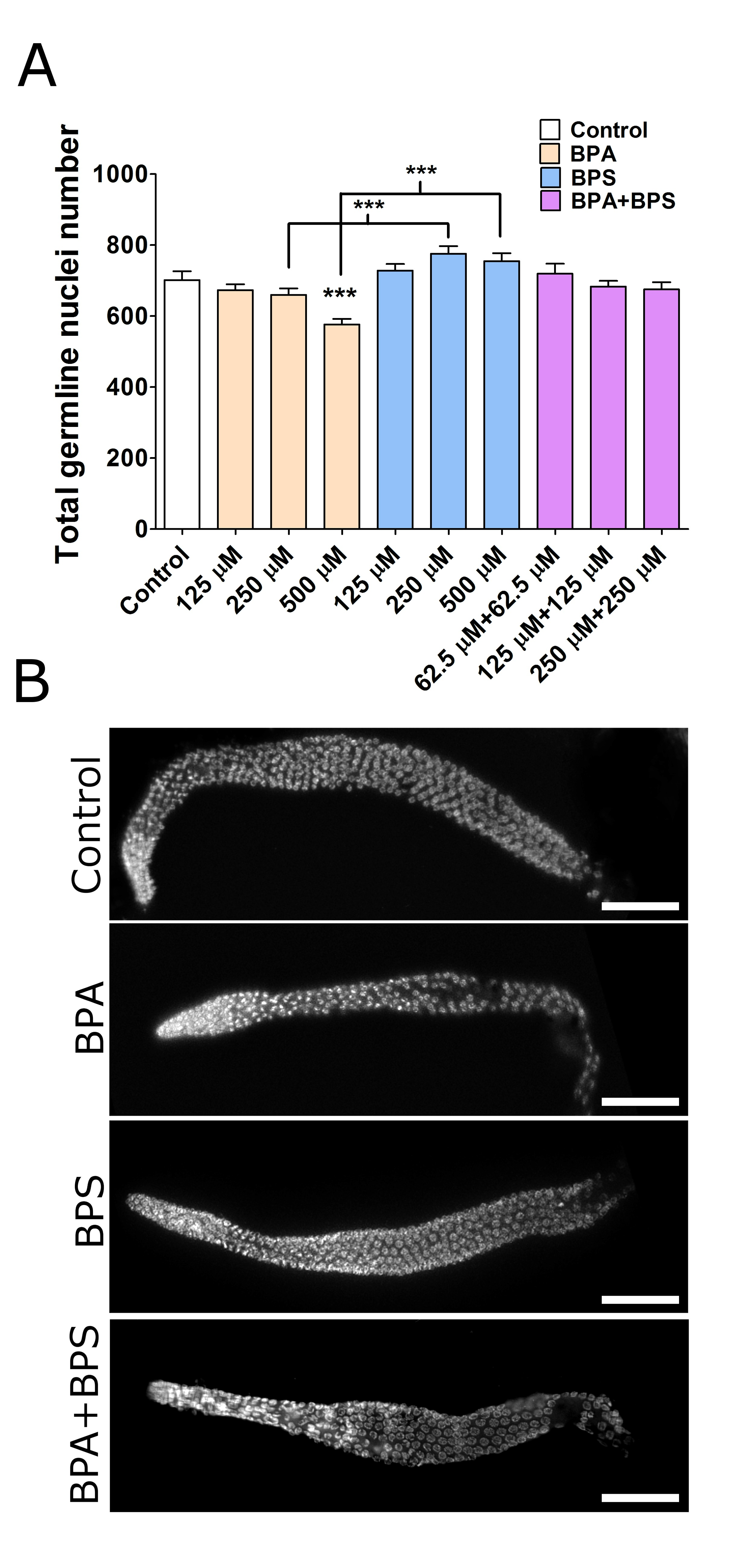

Supplement: S1 Fig — (A) Mean number of germline nuclei in the gonad of worms exposed to vehicle or to the indicated doses of BPA and/or BPS. Error bars represent SEM. More than 5 worms were analyzed per trial and three repeats were performed per treatment group. One-way ANOVA with Tukey’s Post hoc test between control and same treatment with different concentrations. Two-way ANOVA between same concentrion groups of BPA and BPS. ***P<0.001 (B) Low magnification images of DAPI-stained whole-mount gonads from age-matched hermaphrodites exposed to vehicle control (0.1% ethanol), 500 μM BPA, 500 μM BPS or to their mixure (250μM BPA+250μM BPS). (Scale bar, 20 μm). (TIF) [file pgen.1006223.s003.tif]

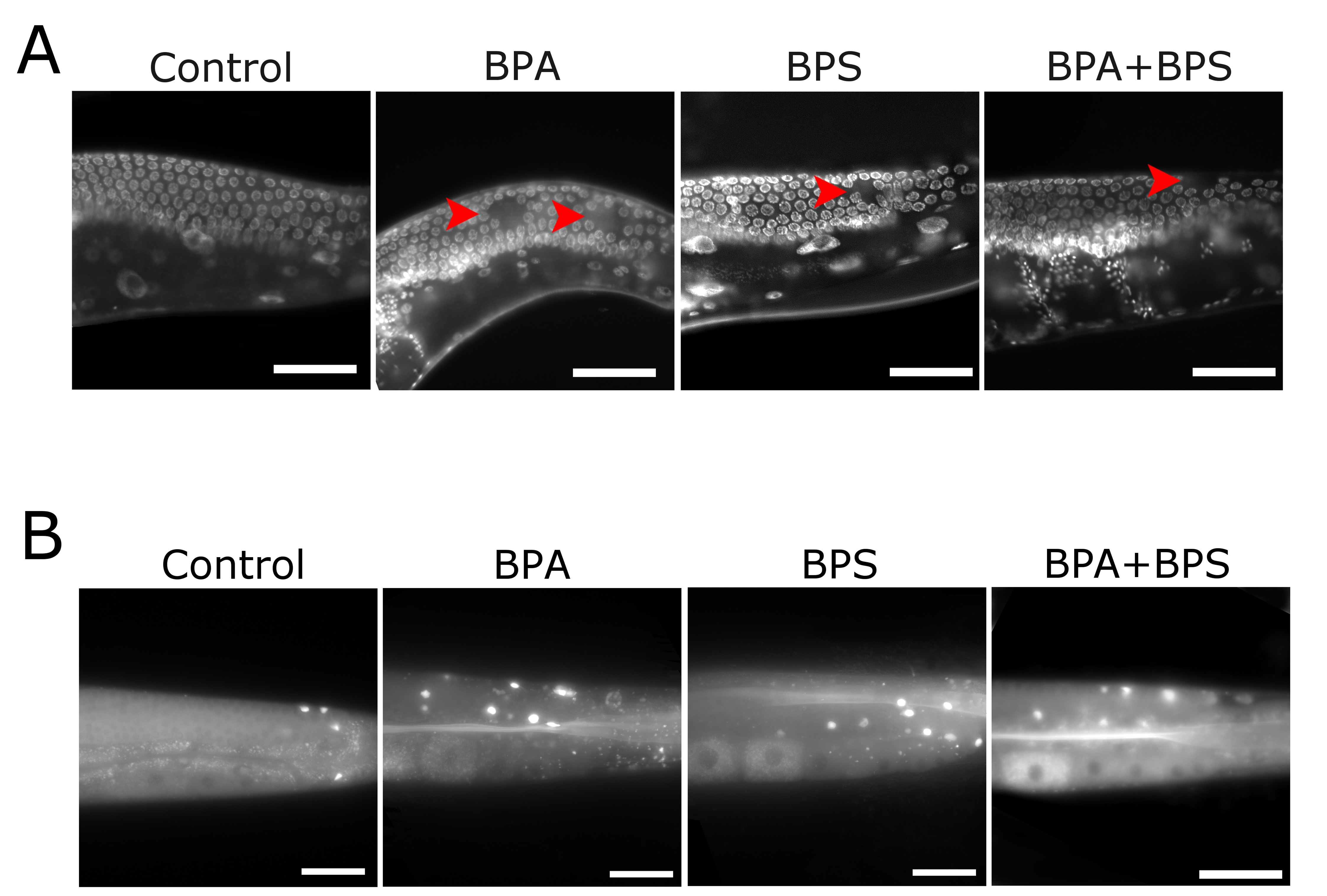

Supplement: S2 Fig — (A) Low magnification images of DAPI-stained gonads from age-matched hermaphrodites exposed to vehicle control (0.1% ethanol), 500 μM BPA, 500 μM BPS or to their mixture (250μM BPA+250 μM BPS). Red arrows indicate the areas with gaps in gonads which refer to the germ cell loss, N = 20 worms per trial performed in triplicate (Scale bar, 25 μm). (B) Low magnification images of acridine orange-stained apoptotic nuclei in the gonads from age-matched hermaphrodites exposed to vehicle control (0.1% ethanol), 125 μM BPA, 125 μM BPS or to their mixture (62.5 μM BPA+62.5 μM BPS). The highlight spots in each gonad are apoptotic nuclei, N = 20 worms per trial, four repeats per each group (Scale bar, 25 μm). (TIF) [file pgen.1006223.s004.tif]

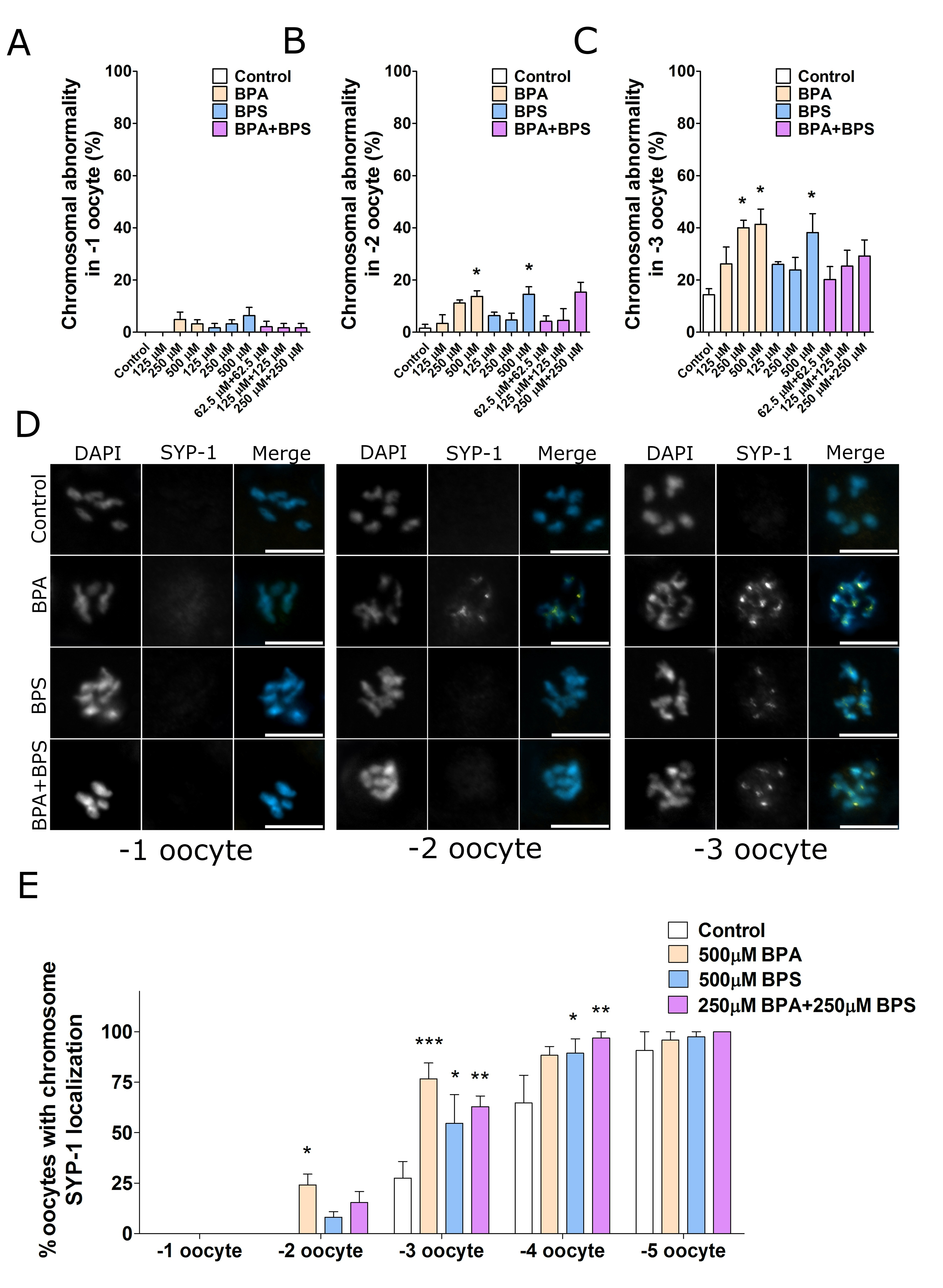

Supplement: S3 Fig — (A-C) The frequency of -1 to -3 oocytes with chromosomal abnormalities in worms exposed to vehicle control (0.1% ethanol) or to the indicated doses of BPA and/or BPS. The oocyte closest to the spermatheca is referred to as the -1 oocyte. Error bars represent SEM. N = 20 worms per trial performed in triplicate (D) DAPI and SYP-1-stained chromosomes in -1 to -3 oocytes from age-matched hermaphrodites exposed to vehicle control (0.1% ethanol), 500 μM BPA, 500 μM BPS or to their mixture (250 μM BPA+250 μM BPS). Six intact bivalents are observed in the control (vehicle) group. In contrast, chromosomes in oocytes are less defined and organized in the worms treated with Bisphenols and show detectable SYP-1 (green) staining (Scale bar 3 μm). (E) Histogram depicts the frequency of observing SYP-1 expression in -1 to -5 oocytes of worms exposed to vehicle control (0.1% ethanol), 500 μM BPA, 500 μM BPS or to their mixture (250 μM BPA+250 μM BPS). Error bars represent SEM. N = 10 worms per trial performed in triplicate. One-way ANOVA with Tukey’s Post hoc test between control and same treatment with different concentrions. Two-way ANOVA between same concentrion groups of BPA and BPS. *P<0.05, **P<0.01 and ***<0.001. (TIF) [file pgen.1006223.s005.tif]

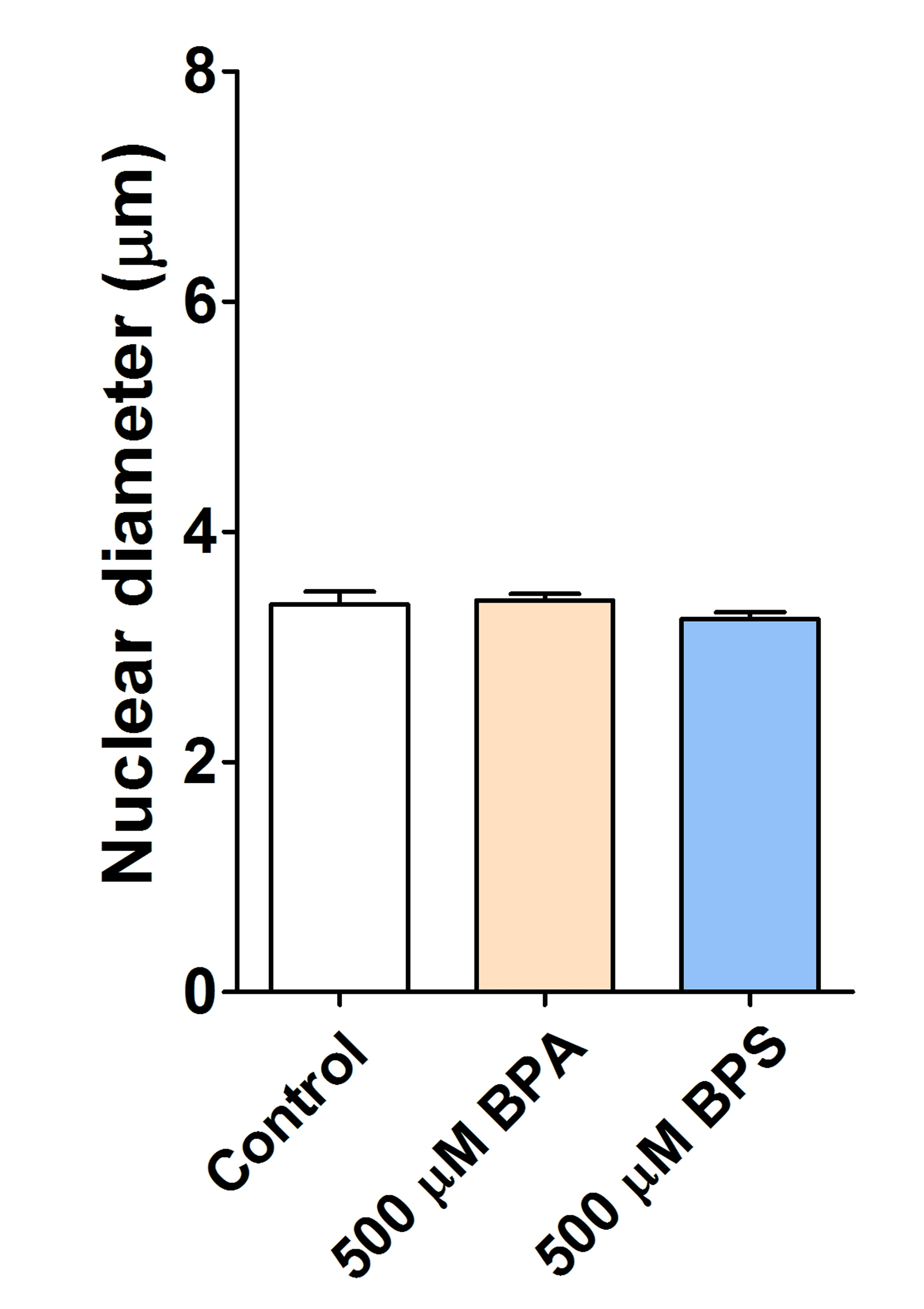

Supplement: S4 Fig — The average diameter of germline nuclei in the mitotic zone from worms exposed to vehicle control (0.1% ethanol), 500 μM BPA or 500 μM BPS was measured. No statistically significant difference was observed between treatment groups and control. Error bars represent SEM. N = 6 worms per trial performed in triplicate. Two-tailed student’s t-test between control and each treatment group. (TIF) [file pgen.1006223.s006.tif]

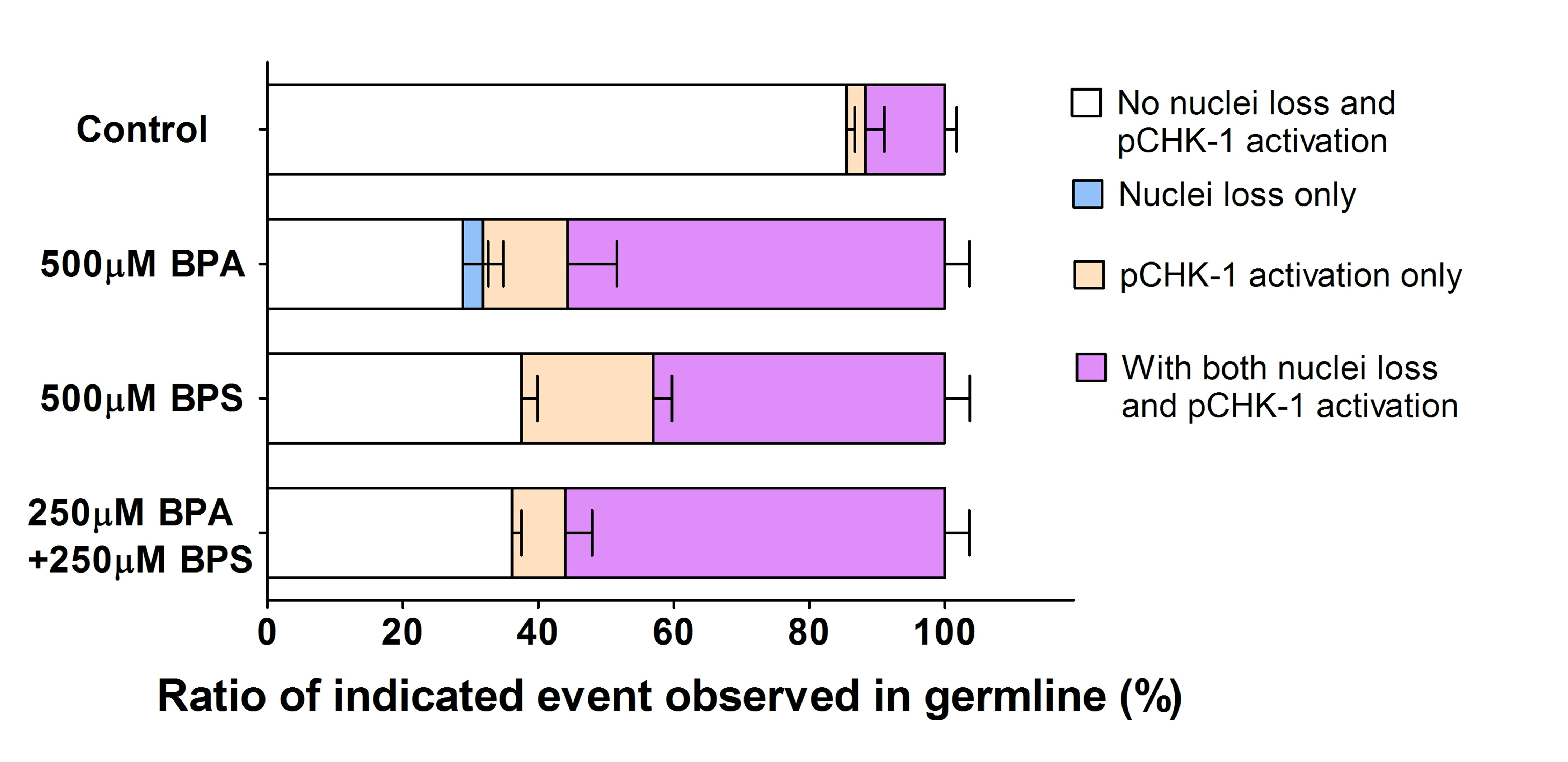

Supplement: S5 Fig — Two events in the germline, nuclear loss and pCHK-1 activation, from worms exposed to vehicle (0.1% ethanol), 500 μM BPA, 500 μM BPS and their mixture were recorded simultaneously. The concordance of pCHK-1 activation and the presence of gaps in all treatments was analyzed by Spearman’s rank correlation test. The correlation coefficient = 0.800, p<0.001, indicating a high positive correlation between these two events. Error bars represent SEM. N = 10 worms per trial, three repeats per treatment group. (TIF) [file pgen.1006223.s007.tif]

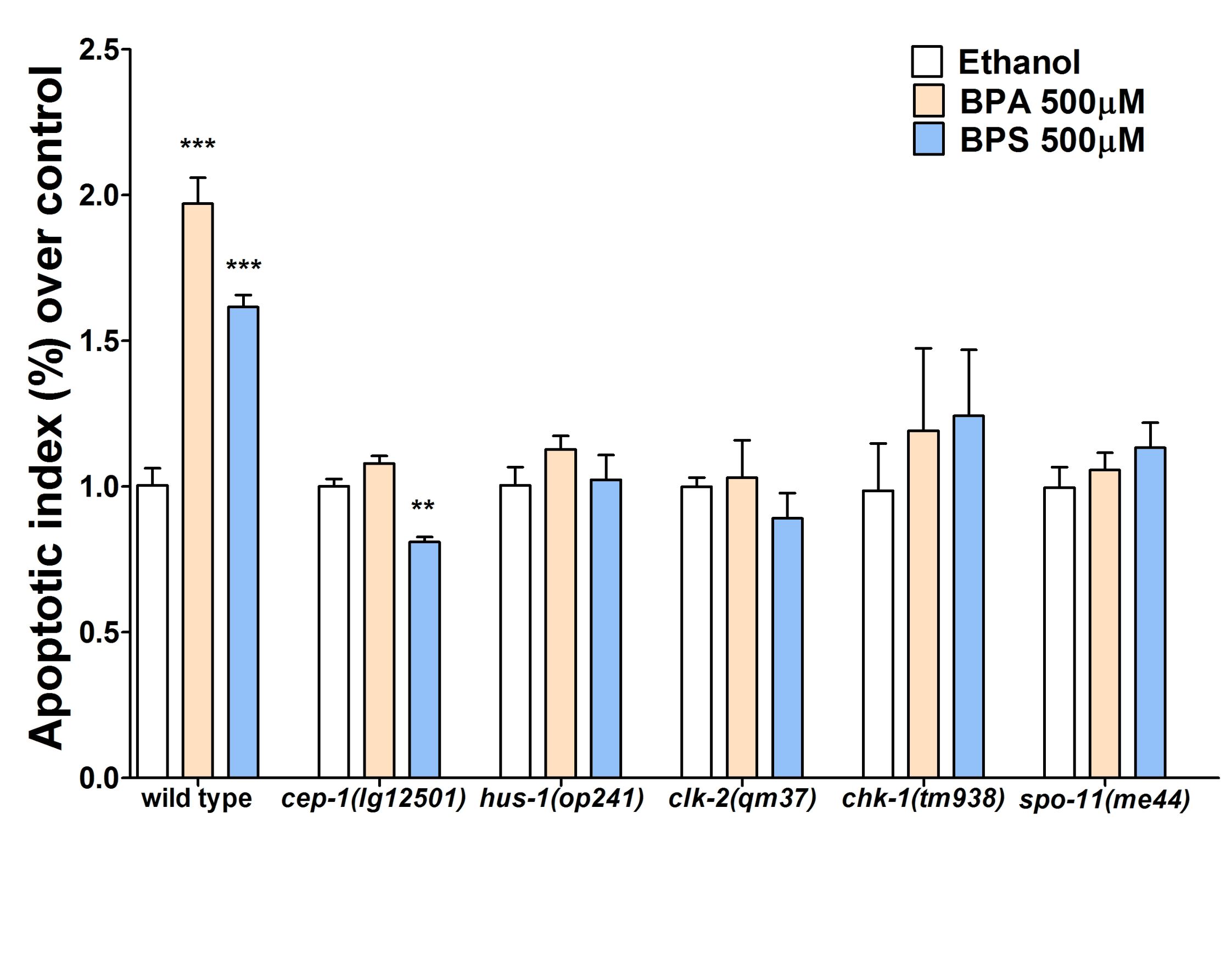

Supplement: S6 Fig — The normalized apoptotic values per 100 pachytene nuclei relative to ethanol control were measured in 6 genetic backgrounds: N2 (wild type), cep-1, hus-1, clk-2, spo-11 and chk-1. In all mutant backgrounds, no statistical difference was observed between BPA or BPS and control except for cep-1 where a small reduction of apoptotic numbers was observed following BPS exposure. N = 13–24 worms per trial performed in triplicate or quintuplate (for N2 and spo-11 backgrounds). Two-tailed student t-test based analysis, *P<0.05, ***P<0.001. (TIF) [file pgen.1006223.s008.tif]

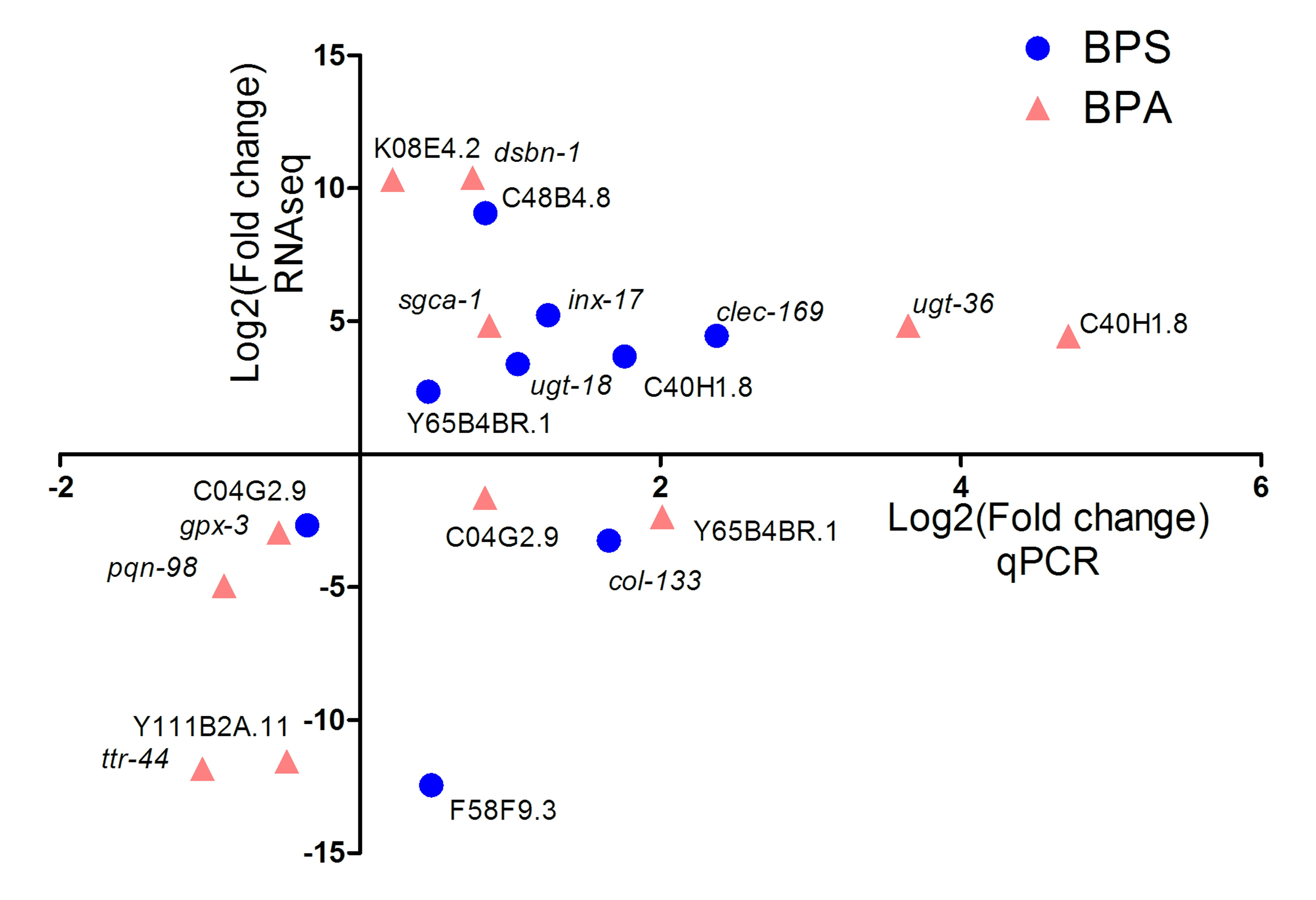

Supplement: S7 Fig — Correlation of RNA-seq (y axis) with qPCR data (x axis) using the log2 fold change measure of a total to 20 genes/BPA or BPA exposure tests were analyzed. 16 out of 20 genes showed directional consistency. The Spearman’s rank correlation coefficient = 0.645, p = 0.002, shows a positive correlation between the two data sets. (TIF) [file pgen.1006223.s009.tif]

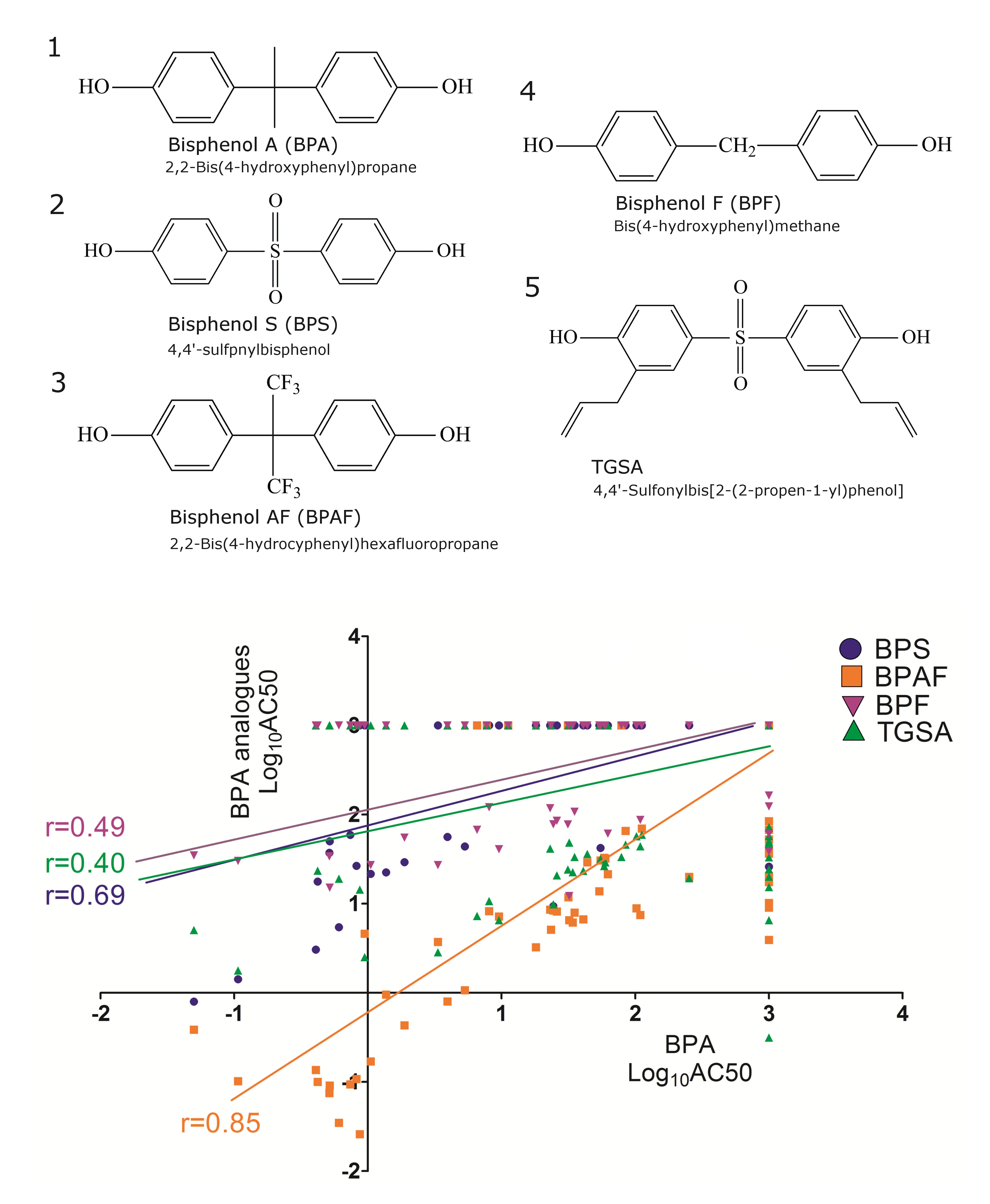

Supplement: S8 Fig — Biological similarities were determined by the Pearson correlation coefficient, r value, between BPA and each of its analogues using the log10AC50 across 126 Toxcast assays. Based on the proximity of r value toward 1, the biological similarity to BPA among four tested analogues is BPAF>>BPS>BPF>TGSA. (TIF) [file pgen.1006223.s010.tif]

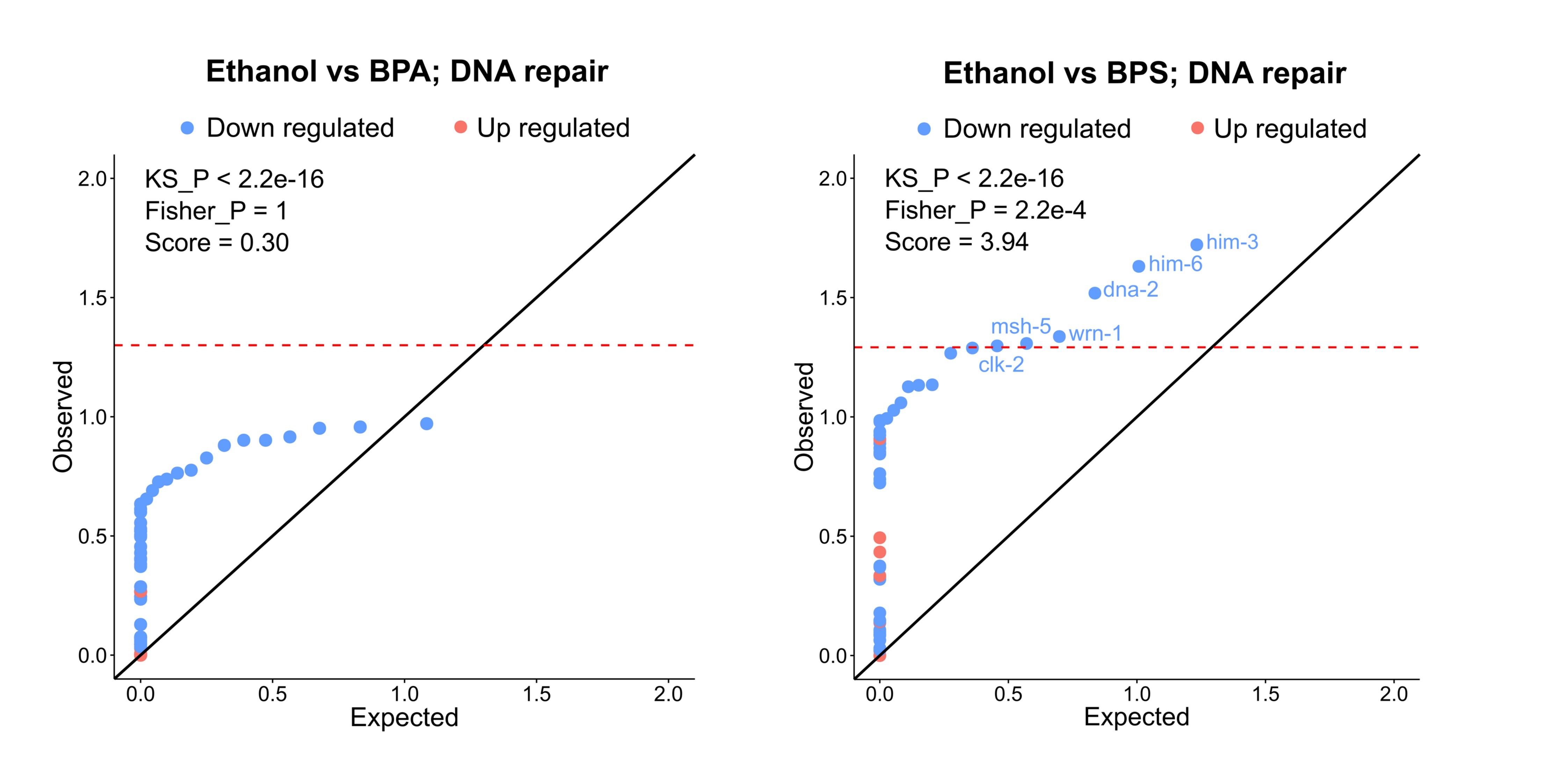

Supplement: S9 Fig — We evaluated the overall pattern of differential expression among these DNA repair genes using one-sided Kolmogorov-Smirnov (KS) test and Fisher’s exact test summarized into an enrichment score, defined as log10((PKS+PFisher)/2) to allow comparison of the degrees of enrichment between BPA and BPS. The P value distributions of the DNA repair genes in the BPA and BPS experiments are visualized using q-q plots, where the quantiles of the log-transformed p-values of DNA repair genes (observed) are plotted against the quantiles of the log-transformed P-values of all genes (expected). A shift of the P value distribution to the upper left direction in the q-q plots indicates an overall enrichment of small P values in the DNA repair genes compared to random genes. Dashed red line indicates cut-off P value of P = 0.05. (TIF) [file pgen.1006223.s011.tif]
