# Supplementary material for: Exposure to the BPA-Substitute Bisphenol S Causes Unique Alterations of Germline Function
Source: PLoS Genet. 2016 Jul 29;12(7):e1006223. doi: 10.1371/journal.pgen.1006223 (PMC4966967; doi:10.1371/journal.pgen.1006223)
Supplement: S1 Data — (ZIP) [file pgen.1006223.s012.zip › Table S1.docx]

| **Sample** | **Sample weight(g)** | **BPs Conc. (ug/ml)** | **Sample Volume (ul)** | **Final**  **Conc. (ug/g)** | **Mean**  **Conc. (ug/g)** | |
| --- | --- | --- | --- | --- | --- | --- |
| **BPA 0.125 (1)** | **0.2348** | **0.384** | **50** | **0.200** | **0.228** | |
| **BPA 0.125 (2)** | **0.2274** | **0.435** | **50** | **0.233** |  | |
| **BPA 0.125 (3)** | **0.1301** | **0.325** | **50** | **0.250** |  | |
| **BPA 0.25 (2)** | **0.2333** | **1.064** | **50** | **0.556** | **0.681** | |
| **BPA 0.25 (3)** | **0.1125** | **0.559** | **50** | **0.606** |  | |
| **BPA 0.25 (4)** | **0.0988** | **0.767** | **50** | **0.880** |  | |
| **BPA 0.5 (1)** | **0.0389** | **0.668** | **50** | **2.094** | **1.889** | |
| **BPA 0.5 (3)** | **0.0836** | **1.409** | **50** | **2.050** |  | |
| **BPA 0.5 (4)** | **0.0816** | **1.019** | **50** | **1.521** |  | |
|  |  |  |  |  |  | |
| **BPS 0.125** | **Below the LOQ (0.16ug/g)** | | | | |  |
| **BPS 0.5 (2)** | **1.9253** | **2.366** | **30** | **0.198** | **0.208** | |
| **BPS0.5 (3)** | **1.9253** | **2.428** | **30** | **0.203** |  | |
| **BPS 0.5 (1)** | **1.9253** | **2.667** | **30** | **0.223** |  | |
| **BPS 0.5 (1)** | **2.0594** | **5.093** | **30** | **0.398** | **0.386** | |
| **BPS 0.5 (2)** | **2.0594** | **4.855** | **30** | **0.380** |  | |
| **BPS0.5 (3)** | **2.0594** | **4.859** | **30** | **0.380** |  | |

GC-MS result

Mean value:

BPA 0.125mM—0.228ug/g BPS 0.125mM—lower than 0.16ug/g

BPA 0.25mM—0.681ug/g BPS0.25mM—0.208ug/g

BPA 0.5mM—1.889ug/g BPS0.5mM—0.386ug/g
